# Effect of magnitude and variability of energy of activation in multisite ultrasensitive biochemical processes

**DOI:** 10.1371/journal.pcbi.1007966

**Published:** 2020-08-06

**Authors:** Leonila Lagunes, Lee Bardwell, German A. Enciso

**Affiliations:** 1 Developmental and Cell Biology Department, University of California Irvine, California, United States of America; 2 Mathematics Department, University of California Irvine, California, United States of America; University of Pittsburgh, UNITED STATES

## Abstract

Protein activity is often regulated by ligand binding or by post-translational modifications such as phosphorylation. Moreover, proteins that are regulated in this way often contain multiple ligand binding sites or modification sites, which can operate to create an ultrasensitive dose response. Here, we consider the contribution of the individual modification/binding sites to the activation process, and how their individual values affect the ultrasensitive behavior of the overall system. We use a generalized Monod-Wyman-Changeux (MWC) model that allows for variable conformational free energy contributions from distinct sites, and associate a so-called activation parameter to each site. Our analysis shows that the ultrasensitivity generally increases as the conformational free energy contribution from one or more sites is strengthened. Furthermore, ultrasensitivity depends on the mean of the activation parameters and not on their variability. In some cases, we find that the best way to maximize ultrasensitivity is to make the contribution from all sites as strong as possible. These results provide insights into the performance objectives of multiple modification/binding sites and thus help gain a greater understanding of signaling and its role in diseases.

## Author summary

Multisite protein modification is ubiquitous in gene regulation and signal transduction, often in the form of multisite phosphorylation. Many models of multisite ultrasensitivity are available in the literature, but they usually assume that all sites contribute equally to the activation of the multisite target. In this work, we relax this assumption and carry out computational and mathematical analysis of a multisite system in which the conformational free energy contribution varies across sites. We find that the ultrasensitivity of the system tends to increase (with some exceptions) when the conformational free energy contributed by any given site is strengthened. Our analysis predicts that all active sites should have approximately the same conformational free energy contribution, a property observed in proteins with unstructured modification domains and bulk electrostatics. We were also able to predict from first principles an energy range of -2 to -4 kcal/mol per site that effectively maximizes ultrasensitive behavior. This prediction is consistent with experimental measurements in phosphorylation sites. Another strategy predicted by some of our models is to select a subset of the sites and activate them uniformly, while silencing other modification sites in the protein. This strategy is also observed experimentally in many multisite phosphorylation proteins.

## Introduction

Cellular systems rely heavily on signal transduction and environmental sensing pathways to successfully respond to internal and external environmental signals and conditions. To regulate signal transduction cascades, mammalian cells use ligand binding or post-translational modifications (PTMs) such as protein phosphorylation, methylation or ubiquitination. Several forms of disease can arise when there are defects in signal transduction pathways, including cancer, diabetes, and heart disease.

Many proteins regulated by ligands or PTMs are multisite proteins, that is, they have multiple sites on which they can be modified or where a ligand can bind. For example, activation of mitogen activated protein kinases requires phosphorylation on two sites [1], and the hemoglobin tetramer has four sites where oxygen can bind ([2] and references therein). In fact, some proteins have more than 150 modification sites [3]. Ligand binding/PTMs can either promote or inhibit protein activity through conformational changes [4, 5], and can influence the target’s enzymatic activity, location, stability, or interactions with other macromolecules [6].

A common role for multisite modifications lies in the creation of switch-like, or ultrasensitive, dose response curves [7–9]. These are positive, monotonically increasing, sigmoidal functions that have two important properties: first, they respond minimally to low levels of input; second, once the input is sufficiently large, they switch from a low output to a near maximal output in response to a relatively small increase in the input. In other words, ultrasensitive systems can both filter out low-level noise and respond with a high gain over an appropriate range of input. Ultrasensitivity has important roles in signal transduction, and a widely-studied problem is how to implement such dose responses using common biochemical reactions [2].

A classical 1965 model by Monod, Wyman and Changeux (MWC) [10] uses multisite modifications to create ultrasensitive responses. This model remains highly influential today [11–14]. In the MWC model, the target molecule/receptor can be in either an active (relaxed) conformation or an inactive (tense) conformation, and ligand binding/PTMs can influence the probability that the target is in one state or the other. One way to envision this is that ligand binding promotes a conformational change that flips the target from inactive to active (or from active to inactive in the case of an inhibitory ligand). In the MWC model, this is equivalent to the point of view that the ligand binds preferentially to the active conformation, a phenomenon known as conformational selection. In multisite MWC models, there are multiple binding sites for ligand, each of which can be either empty or bound. Such models exhibit cooperativity in ligand binding, as the binding of some ligands to the target will promote flipping to the active state, and in the active state, all binding sites have a higher affinity for ligand. In other words, the presence of ligand increases the probability of the receptor existing in the state with higher ligand affinity, thereby increasing the probability of the next ligand binding. In addition to ‘cooperativity’, the term ‘allostery’ is frequently used in conjunction with MWC models, and refers to the effect that one ligand binding to the target has on additional “distant” binding sites in the same molecule, as well as to the effect that ligand binding has on the conformational change that activates the target. The concepts of ultrasensitivity, allostery and cooperativity are important not only in understanding the logic of cellular regulation, but also with regard to disease pathology and drug discovery [15].

Classical mathematical models of allostery and cooperative ligand binding, such as the MWC model, were based on observations of cooperativity between symmetric subunits of oligomeric proteins, such as hemoglobin (a tetramer), threonine deaminase (also a tetramer) and aspartate transcarbamylase (a hexamer) [16]. Given that the molecules under study consisted of multiple identical or very similar subunits, it made sense to treat all binding sites as identical. More recently, however, the concept of allostery has been expanded to include monomeric proteins, where binding of a ligand at one site can result in modulation of function or binding at a (perhaps) distant site in the same polypeptide chain [17]. For instance, binding or modification events occurring in an intrinsically-disordered segment of a protein can promote its folding, and this can be communicated to an adjacent segment, with the net effect that a coupled folding-and-binding event or PTM in one region of the protein influences subsequent interactions or modifications at a distant site(s) within the same monomer [18]. Yet another example is hetero-oligomers that display cooperativity such as the ATPase rings in the proteasome and CCT chaperonin complex [19–22]. In such cases, there is no reason to expect that binding/modification sites will be identical, or that they will make identical contributions to the underlying conformational change once bound/modified.

In the current paper, we set out to explore multisite systems in which the modification of some sites may have a stronger effect on the induced conformational change than the modification of other sites. To do this, we generalize the classical MWC system and assign different parameters to different sites. We aim to determine what combinations of parameters lead to a high level of ultrasensitivity. Each site *i* is assigned an activation parameter *c*_*i*_, generalizing the parameter *c* in the original formulation of the MWC model. Small values of the parameter *c*_*i*_ correspond to a strong ability for the *i*-th site to activate the protein. One can also associate to each site a corresponding conformational free energy contribution, that is, the difference in the Gibbs energy function associated to the site *i*, Δ*G*_*i*_ = *rt* ln(*c*_*i*_). In other words, *rt* ln(*c*_*i*_) is the site-specific free energy contribution to tense-to-relaxed flipping from ligand binding at site *i*. Notice that the conformational free energy contribution Δ*G*_*i*_ is negative number when the activation parameter *c*_*i*_ is less than one, and becomes more negative as *c*_*i*_ approaches 0. Also note that a large negative Δ*G*_*i*_ (and hence a small *c*_*i*_) corresponds to a strong conformational free energy contribution, which will promote flipping to the active state. In contrast, if *c*_*i*_ > 1, then Δ*G*_*i*_ will be positive, meaning that the modification does not promote flipping to the relaxed/active state but instead makes it more likely that the target will stay in the tense state.

Our main results can be summarized as follows. First, making the conformational free energy contribution associated with ligand binding to a single site i more favorable (that is, making this free energy change more negative, which is equivalent to making the activation parameter *c*_*i*_ smaller) has a strong tendency to increase the ultrasensitivity of the system, as measured by its associated Hill coefficient *H*. This effect is not guaranteed as there are some exceptions, especially for low values of the number of sites *n*, but it holds in most circumstances and under several orders of magnitude for the parameters in the system.

Second, for a fixed number of sites *n*, each of which is at least moderately active, one can calculate the average of the activation parameters and get a good approximation of the Hill coefficient of the system by assuming that all sites have this average activation parameter. That is, the Hill coefficient is approximately independent on the variability of the parameters *c*_*i*_, only on their mean value.

Third, we find that when the cost of site maintenance is taken into account, one can obtain an optimal ultrasensitive behavior by focusing on a subset of the sites. The strategy is to have a subset of the sites be equally active, and all other sites have a low or negligible conformational free energy contribution. This prediction has been indeed observed in a number of experimental systems, where only a subset of the sites have the ability to activate the protein. In addition, we demonstrate that there are diminishing marginal ultrasensitivity increases in response to conformational free energy contribution improvements, which allows us to predict a maximal effective conformational free energy contribution per site, on the order of -2 to -4 kcal/mol. This prediction follows from first principles of the mathematical model, and it is surprisingly consistent with experimental data for a typical protein phosphorylation site [23–26].

For completeness, the last sections contain a study of ultrasensitive behavior in a non-allosteric multisite model where all sites are independent from each other, applicable in some cases where the MWC allosteric assumptions are not satisfied. It was found that this system has a more complex relation between activation parameters and ultrasensitivity, which was explored both through computations and mathematical analysis.

## Results

### Generalized MWC dose response

**Fig 1:**
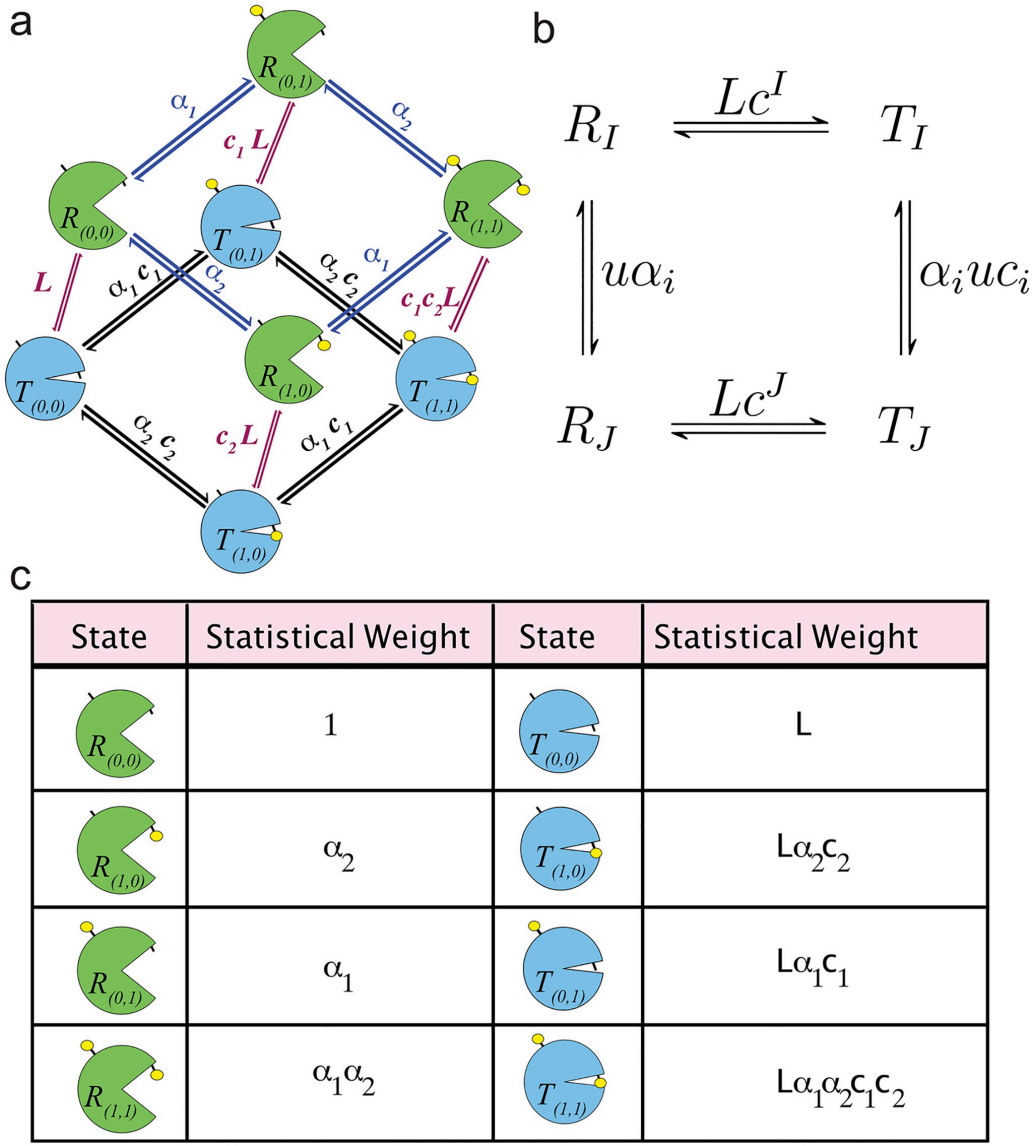
Generalized MWC allosteric model. (a) The figure shows the eight possible states of a target molecule/receptor regulated by the MWC mechanism and containing two sites for ligand binding or post translational modification (i.e., *n* = 2). The four states shown in blue (with closed ‘mouth’) are the tense, inactive states, while the four states shown in green (with open ‘mouth’) are the relaxed, active states. Modification/ligand binding is indicated by the presence of absence of a small yellow ball. The *L*, *α* and *c* parameters are explained in the text. (b) Chemical reaction network demonstrating the possible modified-forms of a receptor with *n* sites, where *I* is the index vector for the modified-form and *J* is the index vector after adding one more modification at site *i*. (c) Table of statistical weights for each state possible with *n* = 2.

In this section, we carry out a generalization of the MWC model to account for different activation parameters at distinct sites. See Enciso and Ryerson [8], where a similar generalization was carried out for protein modification efficiencies. Consider a target molecule with n sites in modified-form *I*, in one of two states, relaxed (*R_I_*) or tense (*T_I_*), where *I* ∈ {0, 1}*^n^* is a binary vector representing the modified-form of the target. In the case of protein activation models, relaxed and tense states correspond to different levels of activity. We assume that all modified-forms in the relaxed (*R*) conformation are active and have the same activity, whereas all modified-forms in the tense (*T*) conformation are inactive, and have the same (low) activity. Under MWC assumptions, the relaxed state has a higher affinity to the ligand than the tense state, this is assumed here for the states *R_I_* and *T_I_*. The unmodified state, $I=\vec{0}=\left(0,0,\cdots ,0\right)$, and the fully-modified state, $I=\vec{1}=\left(1,1,\cdots ,1\right)$, are the two extreme modified-forms, and a total of 2*^n^* modified-forms are possible. The modification of site i on the target will result in modified-form J, where *J* = *I* ∪ {*i*}. In other words J is the modified-form consisting of adding one more modification at site i to the modified-form I. For example, in Fig 1a, a two-site target can be in the relaxed state with no modifications *R*_(0,0)_ and be reversibly modified to *R*_(1,0)_ or *R*_(0,1)_ and subsequently to *R*_(1,1)_. A target in a relaxed state can also flip to the tense state in that form. For instance, *R*_(1,1)_ can flip to the *T*_(1,1)_ state. Similarly, the tense target in modified-form *T*_(1,0)_ can be reversibly modified to *T*_(1,1)_. We call *u* the kinase concentration in the case of multisite phosphorylation and *u* denotes the ligand concentration in the case of ligand binding.

The general system can be described by the chemical reaction network in Fig 1b. The parameter *α*_*i*_ is the microscopic association constant for ligand binding at site *i*. Note that *α*_*i*_ is an affinity, i.e. not a dissociation constant but the inverse thereof. If instead of ligand binding the protein is modified by post-translational modification (phosphorylation, acetylation, etc.), *α*_*i*_ represents the modification efficiency of site *i*, which, for example, will be determined by the relative suitabilty of the site to be phosphorylated by a given kinase and dephosphorylated by a given phosphatase. The parameter *L* is the equilibrium constant between $R_{\vec{0}}$ and $T_{\vec{0}}$. *L* is typically assumed to be greater than 1, so that, in the absence of modification, the tense/inactive state is favored over the relaxed/active state. Indeed, *L* > 9 is required for the target to be less than 10% active in the absence of modification. This network has the property of detailed balance, as the product of the equilibrium constants around any closed cycle of states is 1; this is the same as saying that the net free energy change around any closed cycle of states is 0. For this reason each forward and reverse reaction pair is in equilibrium.

We use the notation $c^{I}=\prod_{I_{i}=1}c_{i}$. Notice that *Lc*^*I*^ is the equilibrium constant between the relaxed (active) state *R*_*I*_ and the tense (inactive) state *T*_*I*_. In this sense, one can think of *c*_*i*_ as the contribution to this equilibrium constant made by each individual site *i*, and Δ*G*_*i*_ as the free energy differential between active and inactive protein contributed by modification at site *i*.

The statistical weights for each state possible when *n* = 2 is listed in Fig 1c. The probability of a state, say *R*_(1,0)_ is defined as the ratio between the statistical weight and the partition function *Z*. In the *n* = 2 case, *Z* = 1 + *α*_1_ + *α*_2_ + *α*_1_ *α*_2_ + *L* + *Lα*_1_ *c*_1_ + *Lα*_2_ *c*_2_ + *Lα*_1_ *α*_2_ *c*_1_ *c*_2_. S1 Fig shows a table of the statistical weights of each modification state when *n* = 3 and the associated *Z*. For general *n*, $Z=1+L+\sum_{i=1}^{n}(\alpha_{i}+L\alpha_{i}c_{i})$.

We will use *u* to represent the concentration of ligand or the concentration of modifying enzyme. If the MWC target molecule is a receptor that is regulated by ligand binding, then *u* is the concentration of ligand. If the MWC target molecule is instead regulated by post-translational modification, then *u* is the concentration/activity of the modifying enzyme. In the latter case, we assume that the modifying enzyme is in steady state with a corresponding demodifying enzyme (e.g. a kinase-phosphatase system), and that both enzymes are far from saturation. Under these assumptions, the dose response relating kinase concentration *u* to the fraction of the target sites that are modified is the same as the dose response relating ligand concentration to the fraction of the target sites that are bound. See [6, 28] for further details. Following a similar analysis to that of Enciso & Ryerson [8], since the system is in detailed balance, for every index *I*,

$$R_{I}u\alpha i=R_{J}\;\text{and}\;R_{I}Lc^{I}=T_{I}$$

Solving for *R*_*I*_ and *T*_*I*_, we can relate *R*_*I*_ to $R_{\vec{0}}$ (relaxed protein with no modifications) by induction as:

$$R_{I}=u^{\left|I\right|}R_{\vec{0}}\alpha^{I}\;\text{and}\;T_{I}=u^{\left|I\right|}R_{\vec{0}}\alpha^{I}Lc^{I}.$$

Note that

$$\begin{array}{cc} \sum_{I}u^{\left|I\right|} & =\sum_{i=0}^{n}\left({}_{i}^{n}\right)u^{i}={\left(u+1\right)}^{n},\\ \sum_{I}c^{I}u^{\left|I\right|} & =\sum_{i=0}^{n}\sum_{|I|=i}c^{I}u^{\left|I\right|}=\sum_{i=0}^{n}u^{i}\sum_{|I|=i}c^{I}=\sum_{i=0}^{n}u^{i}\rho_{i}\left(c\right)=\sum_{i=0}^{n}\rho_{i}\left(uc\right)=\prod_{j=1}^{n}\left(uc_{j}+1\right),\\ \sum_{I}u^{\left|I\right|}\alpha^{I} & =\prod_{i=1}^{n}\left(u\alpha_{i}+1\right)\;\text{and}\;\text{similarly,}\;\sum_{I}u^{\left|I\right|}\alpha^{I}c^{I}=\prod_{i=1}^{n}\left(uc_{i}\alpha_{i}+1\right). \end{array}$$

Here, $\rho_{i}(c)=\sum_{|I|=i}c^{I}$ is the symmetric polynomial in i with entries *c* [8]. For example, consider *n* = 2 with *c* = (*c*_1_, *c*_2_). Here, *ρ*_2_(*c*) = *c*_1_ *c*_2_, *ρ*_1_(*c*) = *c*_1_ + *c*_2_, and *ρ*_0_(*c*) = 1. At various points we are able to rewrite a sum into a product, using the principle that if *x*_*i*_ is a constant for *i* = 1, 2, ⋯, *n*, then $\sum_{I}x^{I}=\prod_{j=1}^{n}\left(x_{j}+1\right)$. For general *n*, the above allows us to write

$$\begin{array}{cc} S_{T} & =\sum_{I}R_{I}+T_{I}=R_{\vec{0}}\sum_{I}u^{\left|I\right|}\alpha^{I}+LR_{\vec{0}}\sum_{I}c^{I}u^{\left|I\right|}\alpha^{I}=R_{\vec{0}}\prod_{i=1}^{n}\left(u\alpha_{i}+1\right)+LR_{\vec{0}}\prod_{j=1}^{n}\left(uc_{j}\alpha_{j}+1\right), \end{array}$$

$$\begin{array}{cc} R_{\vec{0}} & =S_{T}\frac{1}{\prod_{i=1}^{n}\left(u\alpha_{i}+1\right)+L\prod_{i=1}^{n}\left(uc_{i}\alpha_{i}+1\right)}. \end{array}$$

The response of this system is given by the total concentration of relaxed protein, regardless of its level of modifications. That is,

$$f\left(u,c,\alpha \right)=\sum_{I}R_{I}=R_{0}\sum_{I}u^{\left|I\right|}\alpha^{I}=R_{0}\prod_{i=1}^{n}\left(u\alpha_{i}+1\right)=\frac{S_{T}\prod_{i=1}^{n}\left(u\alpha_{i}+1\right)}{\prod_{i=1}^{n}\left(u\alpha_{i}+1\right)+L\prod_{i=1}^{n}\left(uc_{i}\alpha_{i}+1\right)}.$$

That is

$$\begin{array}{c} f\left(u,c,\alpha \right)=\frac{S_{T}}{1+L\prod_{i=1}^{n}\frac{uc_{i}\alpha_{i}+1}{u\alpha_{i}+1}}=S_{T}\omega \left(\eta (u,c,\alpha )\right), \end{array}$$ (1)

where $\eta \left(u,c,\alpha \right)=\prod_{i=1}^{n}\frac{uc_{i}\alpha_{i}+1}{u\alpha_{i}+1}$ and $\omega \left(x\right)=\frac{1}{1+Lx}$. Notice from this functional form when any *c*_*i*_ is equal to 1, it simply multiplies the dose response by one and becomes the same as a system with *n* − 1 sites. This is a nontrivial comment which is not obvious from the system otherwise, but it is biologically intuitive. If a target molecule has weak sites, they only contribute weakly or not at all to increase the Hill coefficient. For fixed parameter values *c* and *α*, we define the maximal response $f^{\infty }(c)=\underset{u\to \infty }{\text{lim}}f(u,c,\alpha )$. A simple calculation shows that $f^{\infty }\left(c\right)=\frac{1}{1+Lc_{1}c_{2}\cdots c_{n}}$ and depends only on *c*_1_, *c*_2_, ⋯, *c*_*n*_ and is independent of *α*. This maximal output value will allow us to normalize response curves across different parameter values in the sections below.

Since the effect of varying the modification parameters *α*_*i*_ was extensively described in Enciso and Ryerson [8], in the majority of the discussion here, we will assume that the *α*_*i*_ are equal to each other, and in fact we can set *α*_*i*_ = 1. To see this, one can re-scale *u* by defining $\bar{u}=u\bar{\alpha }$. The new dose response has the same Hill coefficient as the old system, however the new system satisfies *α*_*i*_ = 1 for all *i*. This helps to better understand the effect of individual activation parameters.

### Computational results on MWC ultrasensitivity

Recall from the previous section that *f*(*u*, *c*, *α*) represents the dose response for the generalized MWC system with parameters *c*_*i*_ and *α*_*i*_, for *i* = 1, 2, ⋯, *n*, where *n* is the number of sites, and *u* is the ligand/enzyme concentration.

In this section we carry out a computational analysis of the dose response and its Hill coefficient, where the parameters *c*_*i*_ are sampled logarithmically. More specifically, log(*c*_*i*_) is chosen with uniform distribution between [10^−4^, 0.9]. The parameter *L* ≥ 1 was fixed, and the parameters *α*_*i*_ were chosen to be identical to each other, $\alpha_{i}=\bar{\alpha }$, here the value of $\bar{\alpha }$ does not affect the Hill coefficient.

We calculated the Hill coefficient H by solving for *EC*_90_ and *EC*_10_ with a standard numerical solver. Here, we solved for *u* such that *f*(*u*, *c*, *α*) − *βf*^∞^(*c*) = 0 for both *β* = 10% and 90%. With both *EC*_10_ and *EC*_90_, we can calculate *H* as

$$\begin{array}{c} H=\frac{ln(81)}{ln(\frac{EC_{90}}{EC_{10}})} \end{array}$$ (2)

derived in [27]. *H* > 1 implies the dose response curve is ultrasensitive, while *H* = 1 implies there is no ultrasensitivity, and *H* < 1 shows negative ultrasensitivity. One can also think of *H* > 1 showing that the dose response has a good switch [28]; the larger the value of H the more ultrasensitive the dose response curve.

**Fig 2:**
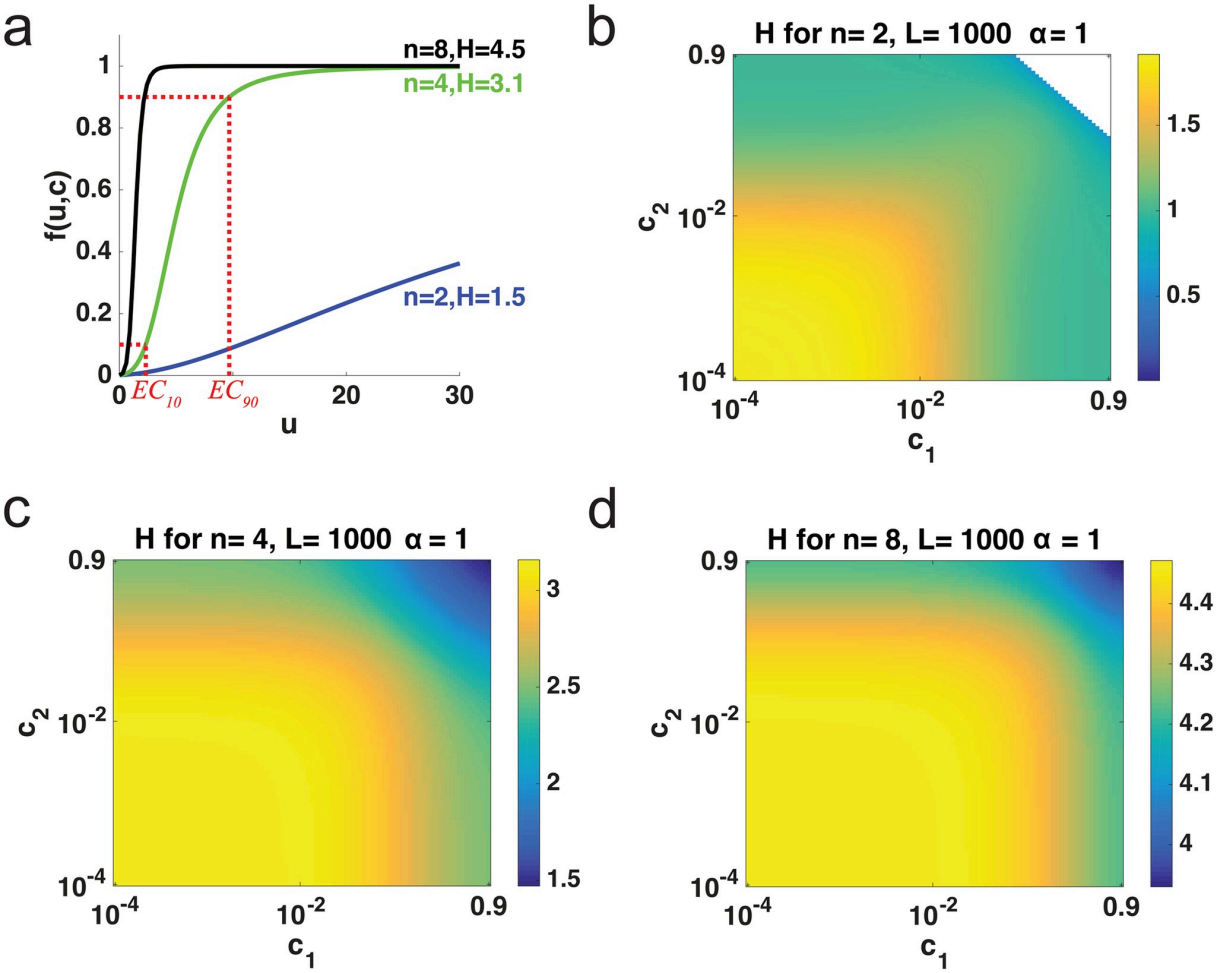
Ultrasensitivity of MWC system. (a) Dose response curve, *f*(*u*, *c*, *α*), when *n* = 2, 4, and 8 for increasing *u* with *c*_*i*_ = 0.01, $\alpha_{i}=\bar{\alpha }=1$ and *L* = 1000. (b-d) Heat maps for *H* when *c*_1_, *c*_2_ ∈ [10^−4^, 0.9] with *L* = 1000 and $\alpha_{i}=\bar{\alpha }=1$ and (b) *n* = 2, (c) *n* = 4, *c*_*i*_ = 0.01 for *i* ≥ 3, similarly with (d) *n* = 8. White indicates undefined *H* values.

Fig 2a displays the dose response curves in this system for *n* = 2, 4, 8, *c*_*i*_ = 0.01, *L* = 1000, and $\alpha_{i}=\bar{\alpha }=1$. These functions show that when all the sites contribute equally, the Hill coefficient tends to increase with the number of sites. In Fig 2b–2d, *c*_1_ and *c*_2_ were increased from 10^−4^ to 0.9 and each *c*_*i*_ = 0.01 for *i* ≥ 3, *α*_*i*_ = 1, and *L* = 1000. In these figures, *H* decreases for increasing *c*_1_ only, suggesting that *H* increases with increasing conformational free energy contribution (recall that larger values of *c* correspond to lower activation contributions). Note that for the *n* = 2 case, there are cases for large values of *c*_1_ where the Hill number is undefined. S2 Fig shows similar data on a linear scale.

In S2 Fig panel e, we show a Monte Carlo approach to study whether *H* is always a decreasing function of *c*_*i*_. By symmetry, we take any individual *c*_*i*_ parameter to be *c*_1_, without loss of generality. To find the proportion of cases where *H* decreases on *c*_1_, we find a numerical approximation to $H_{c_{1}}\left(c\right)$ as follows:

$$H_{c_{1}}\left(c\right)=\frac{\partial H(c)}{\partial c_{1}}\approx \frac{H\left(c_{1}+\Delta x,c_{2},\cdots ,c_{n}\right)-H\left(c_{1},c_{2},\cdots ,c_{n}\right)}{\Delta x},$$

for small Δ*x* to determine if $\frac{\partial H(c)}{\partial c_{1}}<0$. Here, for different values of *L* and *n* with $\alpha_{i}=\bar{\alpha }=1$, for 1000 simulations, we sampled *c*_*i*_ ∈ [10^−4^, 0.9] logarithmically for *i* = 1, 2, ⋯, *n*. The proportion of simulations where *H* decreases on *c*_1_ is almost always one for *n* > 4. For *n* = 2 there are many parameter sets where that is not the case.

**Fig 3:**
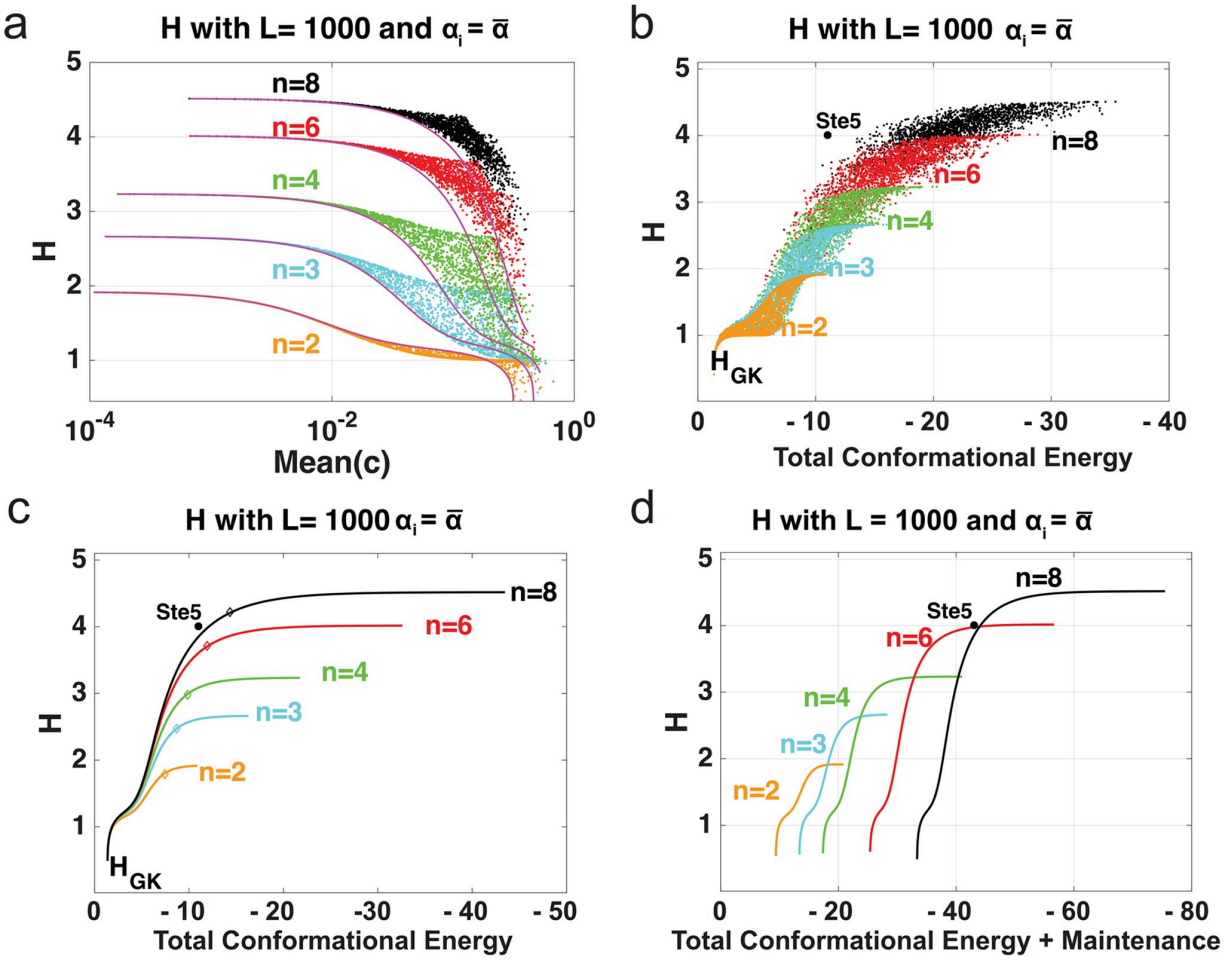
Activation parameters and H in MWC. (a) Scatter plot for H when *c*_*i*_ are independently and logarithmically chosen from the interval [10^−4^, 0.9] (dots) and when *c*_*i*_ are all identical (solid line), *L* = 1000, and $\alpha_{i}=\bar{\alpha }$ for *n* = 2, 3, 4, 6, 8. (b) Scatter plot for *H* for increasing total conformational free energy contribution (Eq 3) when *c*_*i*_ = *c* ∈ [10^−4^, 0.9], $\alpha_{i}=\bar{\alpha }$, and *L* = 1000 for *n* = 2, 3, 4, 6, 8. Asterisk is the approximated *H* for Ste5 from [24]. (c) Scatter plot for *H* for increasing total conformational free energy contribution (Eq 3) when $c_{i}=\bar{c}\in [10^{-4},0.9]$, $\alpha_{i}=\bar{\alpha }$, and *L* = 1000 for *n* = 2, 3, 4, 6, 8. Diamonds represent the knee of the curve. (d) *H* for increasing total conformational free energy contribution where $c_{i}=\bar{c}\in [10^{-4},0.9]$, $\alpha_{i}=\bar{\alpha }$ and *L* = 1000 and with a maintenance cost of 4 kcal/mol per site where *n* = 2, 3, 4, 6, 8. The Ste5 data point is added for illustration purposes with the same maintenance cost for each of the 8 phosphorylation sites.

In Fig 3 we further analyze the effect of varying the activation parameters on the Hill coefficient. In Fig 3a, we sampled a vector $c\in R^{n}$ with entries in the interval [10^−4^, 0.9] logarithmically. This vector of activation parameters has arithmetic mean $\bar{c}$ and coefficient of variation $CV\left(c\right)=\frac{\sigma (c)}{\bar{c}}$. To each vector *c* one can assign a second vector $\hat{c}=\left(\bar{c},\bar{c},\cdots ,\bar{c}\right)$ for which $CV(\hat{c})=0$. After calculating *H* for each case, we can see in Fig 3a, that when there is no variation between *c*_*i*_ (solid line), with parameter values *α*_*i*_ = 1 and *L* = 1000, *H* decreases with increasing mean of *c* for *n* = 2, 3, 4, 6 and 8. Any variation among the *c*_*i*_ does not significantly affect *H* when $\bar{c}<10^{-2}$. For larger values of $\bar{c}$, *H* depends on the variability among the *c*_*i*_ as well as their mean. When *α*_*i*_ is sampled from the range [0.1, 10] the dependence of *H* on $\bar{c}$ is less clearly defined, compared to the case *α*_*i*_ = 1 (see S3 Fig panels a-b).

In Fig 3b, for the same parameter values *c*_*i*_ and *α*_*i*_ = 1 we plot *H* against the total conformational free energy contribution Δ*G*_*tot*_ defined as

$$\begin{array}{c} \Delta G_{tot}=rt\;\text{ln}(\prod_{i=1}^{n}c_{i}) \end{array}$$ (3)

where *r* is the gas constant and *t* is the temperature (traditionally *R* and *T* but labeled as *r* and *t*, respectively, to maintain consistent notation and not be mistaken for the MWC tense and relaxed states). For fixed *n*, as the total conformational free energy contribution increases, ultrasensitivity generally tends to increase, and after some threshold, it tends to level off. To increase ultrasensitivity at that point, a target/receptor cannot profitably utilize more conformational free energy contribution, but instead must evolve more sites. As a concrete example, let us consider an MWC molecule with two sites under selective pressure to increase its ultrasensitivity. Changes to the microscopic modification affinities/efficiencies (i.e., the Δ*G*_*i*_‘s) will either decrease ultrasensitivity (if the changes are unbalanced), or at best leave ultrasensitivity unaltered (if the changes are balanced) [8]. Thus, the only viable options to increase ultrasensitivity are to (a) evolve another site, or (b) strengthen the conformational free energy contribution of the existing sites. At first, significant increases to ultrasensitivity can result from the second option. A mutation that strengthens the conformational free energy contribution of one of the sites will move the molecule up and to the right in the cloud of points for *n* = 2 shown in Fig 3b, with the largest jump coming from strengthening the weakest site. As additional mutations of this type arise and become fixed by natural selection, the molecule will move to the top right of the cloud; here the conformational free energies will be approximately balanced and have magnitudes of approximately -2 to -4 kcal/mol. At this point, substantial improvement to ultrasensitivity (i.e., an increase of the Hill number by greater than 0.5 units) can only arise if the molecule evolves an additional site.

To view this more clearly, consider Fig 3c, which shows ultrasensitivity for increasing values of total conformational free energy contribution where parameter values have been set to $c_{i}=\bar{c}$ and $\alpha_{i}=\bar{\alpha }=1$ and fixed *n* and *L*. In other words, when each *c*_*i*_ is the same, meaning the conformational free energy contribution is the same in all sites, we can see that ultrasensitivity generally increases and eventually levels off as the conformational free energy contribution increases. Fig 3c also helps to make a prediction of the energy that each site optimally contributes, given a total conformational free energy contribution.

Calculating the “knee” of the curve provides a rough estimate of the total conformational free energy contribution required before the ultrasensitivity begins to level off. The knee of a saturating curve is a mathematical definition that captures the point at which the curve is reaching saturation. It is defined in our context as max *a* ≤ *x* ≤ *b*|*Y*_*n*_(*c*) − *ℓ*(*x*)|, where *Y*_*n*_(*x*) is the Hill coefficient curve in Fig 3c for *n* sites, *a*, *b* are the lowest and highest total energy values among the data points for *n* sites respectively, and *ℓ*(*x*) is a secant line joining (*a*, *Y*_*n*_(*a*)) and (*b*, *Y*_*n*_(*b*). For a more detailed explanation, refer to Figure 2b in Ref [29].

The approximated knee of each curve in Fig 3c was found and is depicted with a diamond and listed in Table 1. Notice that the energy for saturation increases roughly linearly with the number of sites. In each case, the amount of energy per site is approximately -2 to -4 kcal/mol.

**Table 1:** Ultrasensitivity at knee.

L	n	H	total cfe	cfe/site
1000	2	1.80	-7.37	-3.69
1000	3	2.47	-8.67	-2.89
1000	4	2.99	-9.80	-2.45
1000	6	3.71	-11.97	-1.99
1000	8	4.21	-14.39	-1.80

Ultrasensitivity as measured by the Goldbeter-Koshland formula described in Eq (2) along with the approximated knee of the curves in Fig 3c for fixed values of *L* and *n*. The knee of curve occurs at a single value of total conformational free energy contribution (cfe) with a Hill number *H*. Parameters $\alpha_{i}=\bar{\alpha }=1$.

This analysis is consistent with some previous experimental findings [23, 24]. The approximated *H* for Ste5 from [24] with *n* = 8 phosphorylation sites is indicated with an asterisk in Fig 3b and 3c. We derive the value -1.6 kcal/mol per site in this system, which is equivalent to a 10-fold affinity increase per site as approximated in [24]. Not only does this data point approximately lie close to the curve for *n* = 8, but in fact it lies close to the knee of the curve when all sites contribute an equal amount, as predicted in the above analysis. The marginal effect of an additional kcal of free energy is dependent on only one other parameter, namely *L* (assuming the *α*_*i*_ are roughly equal to each other). If *L* ranges from 30 to 10,000, the analysis is roughly similar (see S3 Fig panels c-d), and it leads to an energy range of around -2 to -4 kcal/mol per site (see also S1 Table).

There are ways of evaluating ultrasensitivity other than the Goldbeter-Koshland method [27]. In S4 Fig, we measure ultrasensitivity in two additional ways: (1) fitting the dose response curve to the Hill function [9] and (2) Levitzki’s *n*_50_ [30]. We see similar results, thus the qualitative results here do not depend on how ultrasensitivity is measured.

In Fig 3d, similar to Fig 3b, we plot *H* for increasing values of total conformational free energy contribution where now we take into account a maintenance cost for each site, denoted by *M*_*c*_. Such a maintenance cost may arise, for example, if there is rapid turnover of a post-translational modification, as has been observed for phosphorylation-dephosphorylation of some substrates [31, 32]. This type of rapid dynamics in modification-demodification cycles could constitute a non-negligible expenditure of energy for the cell.

The total activation energy including maintenance can be calculated as Δ*G*_*tot*_ + *M*_*c*_ * *n*, where Δ*G*_*tot*_ is given by (Eq 3) and *M*_*c*_ = 4 kcal/mol, which was arbitrarily chosen. In this figure, we assume for simplicity that energy is equally distributed among all sites. Once the cost of maintenance is taken into account, one can see more clearly that for each level of total conformational free energy contribution there is an optimal value of n. For instance, if the total energy is -20 kcal/mol, then the optimal number of sites is *n* = 3; any fewer sites will not have as high ultrasensitivity, while any larger number of sites requires an excessive amount of maintenance. If there are more than four sites in this system, it is beneficial to eliminate or silence the remaining sites. Similar qualitative results can be seen in S5 Fig with different maintenance cost values.

To summarize, in this section we have shown that (1) increasing the conformational free energy contribution at a single site has a strong tendency to increase the ultrasensitivity of the response, with some exceptions, (2) for fixed *n*, the ultrasensitivity depends on the mean of the free energies of activation and very little on their variance, and (3) we estimate from first principles an effective energy range of -2 to -4 kcal/mol per site, which is consistent with experimental data.

### Generalized independent dose response

The assumption of cooperativity between sites plays a role in the ultrasensitive behavior of the dose response curves. However, if we do not assume cooperativity between sites, will we observe the same effect in the previous section on *H*? In this section, we use a non-allosteric model and carry out a similar study as for the generalized MWC model. The proposed model has been used elsewhere [8, 33] but here it is generalized for the first time to have different activation parameters at different sites.

Consider a target molecule with *n* modification sites in modified-form *I* ∈ {0, 1}^*n*^, where we no longer assume that there is cooperativity between sites. The target can be in one of two states, *A*_*I*_ (active) or *B*_*I*_ (inactive) and thus gives 2^*n*^ possible modified-forms.

**Fig 4:**
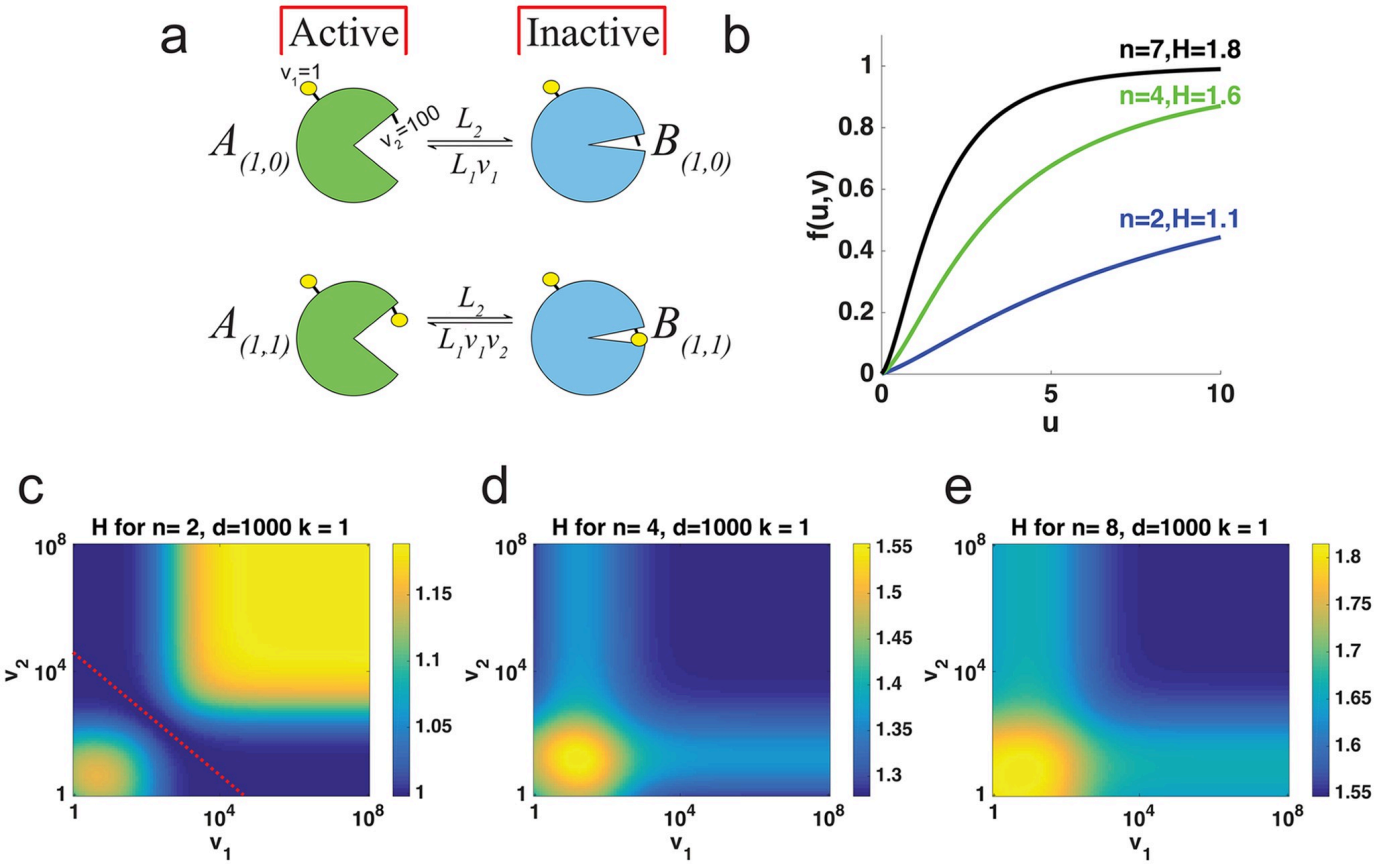
Independent Multisite Modification Model. (a) Target molecule in modified-form *I* can be in the inactive state *B*_*I*_ or active *A*_*I*_ state. (b) Dose response curve, *f*(*u*, *v*) when *n* = 2, 5, 7 for increasing kinase concentration *u* with *v*_*i*_ = 100 and *d* = 1000 (c-e) Heat maps for *H* when *v*_1_, *v*_2_ ∈ [10, 10^8^] with *d* = 1000, and (c) *n* = 2, dashed line used to denote region where $d=\sqrt{v_{1}v_{2}}$. (d) *n* = 4, *v*_*i*_ = 100 for all *i* ≥ 3, similarly with (e) *n* = 7.

The target *S* in modified-form *I* can be described by the chemical reaction in Fig 4a, where *v*_*i*_ > 1 represents the conformational free energy contribution of the *i*-th modification site. Each *v*_*i*_ can also be related with the binding energy of the *i*-th modification site in the MWC model through the formula

$$\begin{array}{c} \Delta G_{i}=-rt\;\text{ln}\left(v_{i}\right). \end{array}$$

That is, the larger the value of *v*_*i*_, the larger the free energy. The parameter *v*_*i*_ can also be thought of as the inverse of $v_{i}=\frac{1}{c_{i}}$ of the activation parameter in the MWC model. For notation purposes, $v^{I}=\prod_{I_{i}=1}v_{i}$.

To obtain the dose response for total target *S*_*T*_ as a function of enzyme concentration *u*, we use mass action kinetics on the chemical reaction below

$$\begin{array}{c} A_{I}\overset{d}{\underset{v^{I}}{⇌}}B_{I} \end{array}$$

As in the MWC model, *d* is the reaction rate constant and is analogous to *L*. The associated differential equation for the active target is:

$$\begin{array}{c} \frac{dA_{I}}{dt}=v^{I}B_{I}-dA_{I}, \end{array}$$ (4)

with conservation of mass equation for the target in modified-form *I*:

$$\begin{array}{c} S_{I}=A_{I}+B_{I}. \end{array}$$

We allow this reaction to reach equilibrium by assuming that this activation/deactivation reaction is much faster than protein modification. This is a reasonable assumption in the case of protein phosphorylation. Solving for steady state of (4),

$$\begin{array}{cc} 0 & =v^{I}\left(S_{I}-A_{I}\right)-dA_{I}=v^{I}S_{I}-A_{I}(v^{I}+d), \end{array}$$

that is

$$\begin{array}{cc} A_{I}\left(v^{I}+d\right) & =v^{I}S_{I} \end{array}$$

and

$$\begin{array}{cc} A_{I} & =\frac{v^{I}}{v^{I}+d}S_{I} \end{array}$$

In order to calculate the activity level of a target molecule in modified-form *I*, we defined the function *Q*_*I*_(*v*), which can be considered to be the fraction of time a protein is active, as

$$A_{I}=\frac{v^{I}}{d+v^{I}}S_{I}\;\text{,}\;\frac{A_{I}}{S_{I}}=\frac{v^{I}}{d+v^{I}}.$$

Hence,

$$\begin{array}{c} Q_{I}\left(v\right)=\frac{v^{I}}{d+v^{I}}. \end{array}$$ (5)

To further understand *Q*_*I*_(*v*), consider the case when *n* = 2. If both sites are modified, then $Q_{\left(1,1\right)}=\frac{v_{1}v_{2}}{d+v_{1}v_{2}}\approx 1$ for large values of *v*_*i*_ relative to *d*. If neither site is modified, then $Q_{\left(0,0\right)}=\frac{1}{d+1}$. In other words, the activity of a protein increases with the amount of modifications. Notice that the activity level will depend on the activation parameters of the specific sites and the overall number of sites modified.

We can also determine the concentration of *S*_*I*_, as a function of enzyme concentration *u*. We can accomplish this by first considering the fraction, *p*_*i*_, that is modified on the *i*-th site, at steady state. Then, from [8], given *k*_*i*_ is the disassociation rate constant of the *i*-th site,

$$\begin{array}{c} p_{i}\left(u\right)=\frac{u}{u+k_{i}}. \end{array}$$ (6)

We assume that the modification states of the different sites are independent of each other, an assumption that is in a sense the opposite of cooperativity. In other words, the modification of one site does not influence the modification of another. This allows to calculate the proportion of target in state *I* as

$$\frac{S_{I}}{S_{T}}=\prod_{I_{i}=1}p_{i}\prod_{I_{i}=0}\left(1-p_{i}\right)=\prod_{I_{i}=1}\frac{u}{u+k_{i}}\prod_{I_{i}=0}(1-\frac{u}{k_{i}+u})=\prod_{I_{i}=1}\frac{u}{u+k_{i}}\prod_{I_{i}=0}\frac{k_{i}}{k_{i}+u},$$

where *S*_*T*_ is the total amount of target molecule. The dose response is calculated as follows:

$$\begin{array}{c} f\left(u,v\right)=\sum_{I}Q_{I}S_{I}=\sum_{I}\frac{v^{I}}{d+v^{I}}\prod_{I_{i}=1}\frac{u}{u+k_{i}}\prod_{I_{i}=0}\frac{k_{i}}{k_{i}+u}. \end{array}$$ (7)

This function has a maximal output value *f*^∞^(*v*), which is found in a similar fashion to the MWC maximal output value by evaluating the limit of *f*(*u*, *v*) as *u* → ∞. Note that for any *I* containing a zero (i.e., any modified-form with at least one site un-modified), $\underset{u\to \infty }{\text{lim}}S_{I}=0$. Only $I=\vec{1}=\left(1,1,\cdots ,1\right)$ will contain a non-zero limit for *S*_*I*_, giving $f^{\infty }\left(v\right)=Q_{\vec{1}}$. This maximal output value can be used to normalize the dose response curves across different parameter values, similar to the MWC system.

### Computational results on independent system ultrasensitivity

For multisite proteins, we can determine the proportion of active target by calculating *f*(*u*, *v*) from the independent system above. In Fig 4b, we plot dose response functions for *n* = 2, 4, and 8 with *v*_*i*_ = 100, *k*_*i*_ = 1 and *d* = 1000.

Similar to the MWC section above, we show how *H* is affected by the activation parameters of individual sites. In Fig 4c–4e, we measure *H* in a similar fashion to that in Fig 3, by solving for *EC*_10_ and *EC*_90_ given the dose response *f*(*u*, *v*) in (Eq 7). Here, *d* = 1000 and *k*_*i*_ = 1 and parameters *v*_1_ and *v*_2_ were sampled with values in [1, 10^8^] logarithmically and each *v*_*i*_ = 100 for *i* > = 3 for *n* = 2, 4, and 8. This implies that *H* does not increase monotonically with increasing *v*_*i*_, and there is a local minimum for low values of *v*_*i*_.

**Fig 5:**
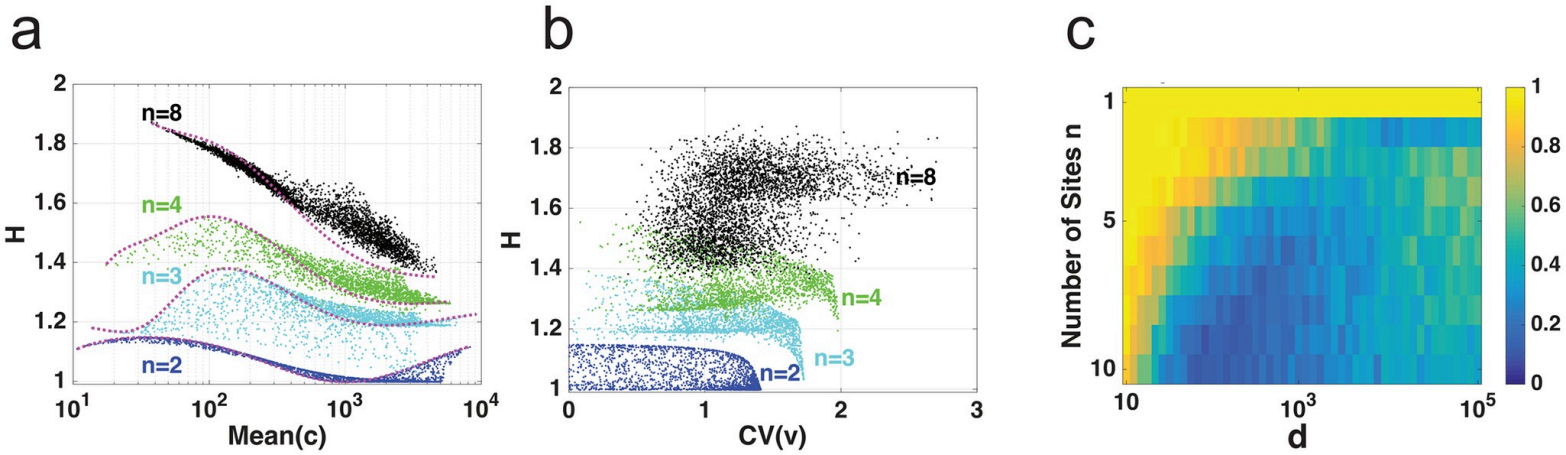
Activation parameters and H in independent model. (a) Scatter plot for H when *v*_*i*_ ∈ [10, 10^4^] (asterisks) and when $v_{i}=\bar{v}$ for *i* = 1, 2, ⋯, *n* (solid curve) for different values of *n*. (b) *H* vs *CV*(*v*) for different values of *n*. (c) Heat map showing the proportion of times *H* increased with increasing *v*_*i*_ as a function of *n* and *d* for *v*_*i*_ ∈ [10, 10^4^] and *k*_*i*_ = 1 with 1000 simulations of randomly chosen *v*_*i*_ ∈ [10, 10^4^].

To determine the effect the variability between parameters *v*_*i*_ has on *H*, we varied parameters *v*_*i*_, measured *H* and compared to when all parameters *v*_*i*_ are equal, similar to the MWC system. In Fig 5a, we sampled a vector with entries from [1, 10^4^], logarithmically. This *v* has an arithmetic mean $\bar{v}$ and coefficient of variation, $CV=\frac{sd(v)}{\bar{v}}$. For each sample, there is a second vector, $\hat{v}=\left(\bar{v},\bar{v},\cdots ,\bar{v}\right)$ such that $CV(\hat{v})=0$. After calculating *H* for each case, we can see in Fig 5a, that when there is no variation between *v*_*i*_ (solid line), *k*_*i*_ = 1 and *d* = 1000, *H* can increase or decrease depending on the mean of *v* for *n* = 2, 3, 4 and 8. Here, we can see that any variation between the *v*_*i*_ may affect *H* (asterisks).

In Fig 5b, we show the same data from Fig 5a but plotting *CV*(*v*) vs *H*. Here we see that *CV*(*v*) has some effect on *H*, regardless of *n*. This is particularly interesting since, contrary to MWC, the variation between *v*_*i*_ affects *H*. We also see that there are values of $\bar{v}$ where *H* increases and values where it decreases. How often is *H* increasing with increasing *v*_1_?

In Fig 5c, similar to S3 Fig panel e, we provide the proportion of simulations where *H* increases with increasing *v*_1_ based on *n* and *d*. The proportion was found in a similar fashion as in the MWC system. Here, we logarithmically sampled *v*_*i*_ ∈ [10, 10^4^] and *k*_*i*_ = 1.

The computational and analytical results described in the section below titled “Independent System Mathematical Analysis” suggest that that $d>\sqrt{v_{1}v_{2}}$ is a biologically reasonable assumption that will give dose response functions where the effect of two modifications is significantly different than the effect of a single modification. Similarly, $d<\sqrt{v_{1}v_{2}}$ gives dose response functions where the effect of a single modification has a similar effect as two modifications, termed “1+” regime. In this “1+” regime we see *H* increasing on *v*. When $d=\sqrt{v_{1}v_{2}}$, we have a dose response function where the effect of one modification is approximately 50% of the effect of two modifications, with no ultrasensitivity (*H* ≈ 1). We can also see that if *d* is slightly past the 50% of max activation, *H* can be maximized by increasing the free activation of energy *v*.

To summarize, in this section we show that (1) ultrasensitivity increases under specific parameter regimes and (2) may depend on the variability between the activation parameters.

### MWC system mathematical analysis

In this section, we provide a mathematical analysis of the generalized MWC system showing that *H*(*c*, *α*) is roughly independent of the variation of *c*. We will show that *H* is essentially a function of $\bar{c}$ and $\bar{\alpha }$. That is, the variability of activation parameters only affects *H* to the extent that it changes the mean, $\bar{c}$. Consider *f*(*u*, *c*, *α*) from Eq (1) and define

$$\bar{\alpha }=\frac{\alpha_{1}+\alpha_{2}+\cdots +\alpha_{n}}{n}\;\text{,}\;\Delta \alpha =\left(\alpha_{1}-\bar{\alpha },\alpha_{2}-\bar{\alpha },\cdots ,\alpha_{n}-\bar{\alpha }\right)\;,$$

$$\bar{c}=\frac{c_{1}+c_{2}+\cdots +c_{n}}{n}\;\text{,}\;\Delta c=\left(c_{n}-\bar{c},c_{2}-\bar{c},\cdots ,c_{n}-\bar{c}\right).$$

For notation purposes, let $\hat{c}=\left(\bar{c},\bar{c},\cdots \bar{c}\right)\in R^{n}$ and $\hat{\alpha }=\bar{\alpha },\bar{\alpha },\cdots ,\bar{\alpha })\in R^{n}$. Recall that given a *C*^2^ function *f* such that $\frac{\partial f}{\partial x}\left(a,b\right)=\frac{\partial f}{\partial y}\left(a,b\right)=0$, it holds that

$$\begin{array}{c} f(x,y)=f(a,b)+o(x-a,y-b). \end{array}$$

We will use this to show that

$$\begin{array}{c} H\left(c,\alpha \right)=H\left(\hat{c},\hat{\alpha }\right)+o\left(\Delta c,\Delta \alpha \right). \end{array}$$

This formula demonstrates in particular that *H* essentially does not vary if the mean of *c* is preserved, as illustrated in Fig 3a.

**Proposition 1** $H\left(c,\hat{\alpha }\right)=H\left(\hat{c},\hat{\alpha }\right)+o\left(\Delta c\right)$

*Proof*: For simplicity, assume *S*_*T*_ = 1 and assume for now that *u* and $\hat{\alpha }$ are fixed. By the approximation of the geometric mean using the arithmetic mean, we have

$$\begin{array}{cc} {\left(\prod_{i=1}^{n}uc_{i}\bar{\alpha }+1\right)}^{1/n} & =\frac{1}{n}\sum_{i=1}^{n}\left(uc_{i}\bar{\alpha }+1\right)+o\left(\Delta c\right)=\left(u\bar{c}\bar{\alpha }+1\right)+o\left(\Delta c\right). \end{array}$$

Taking the *n*-th power, $\prod_{i=1}^{n}\left(uc_{i}\bar{\alpha }+1\right)={\left(u\bar{c}\bar{\alpha }+1\right)}^{n}+o\left(\Delta c\right).$ Let *M* > 0 such that |*ω*(*x*) − *ω*(*y*)| ≤ *M*|*x* − *y*| for all *x*, *y* > 0. Then,

$$\begin{array}{cc} |f\left(u,c,\hat{\alpha }\right)-f\left(u,\hat{c},\hat{\alpha }\right)| & \leq M|\eta \left(u,c,\hat{\alpha }\right)-\eta \left(u,\hat{c},\hat{\alpha }\right)|\\ & =M|\prod_{i=1}^{n}\frac{uc_{i}\bar{\alpha }+1}{u\bar{\alpha }+1}-\prod_{i=1}^{n}\frac{u\bar{c}\bar{\alpha }+1}{u\bar{\alpha }+1}|\\ & =\frac{M}{{\left(u\bar{\alpha }+1\right)}^{n}}|\prod_{i=1}^{n}\left(uc_{i}\bar{\alpha }+1\right)-{\left(u\bar{c}\bar{\alpha }+1\right)}^{n}|=o\left(\Delta c\right). \end{array}$$

Then $f\left(u,c,\hat{\alpha }\right)=f\left(u,\hat{c},\hat{\alpha }\right)+o\left(\Delta c\right)$. It follows that

$$\begin{array}{c} EC_{10}\left(c,\hat{\alpha }\right)=EC_{10}\left(\hat{c},\hat{\alpha }\right)+o\left(\Delta c\right)\;\text{and}\;EC_{90}\left(c,\hat{\alpha }\right)=EC_{90}\left(\hat{c},\hat{\alpha }\right)+o\left(\Delta c\right). \end{array}$$

Thus,

$$\begin{array}{c} H\left(c,\hat{\alpha }\right)=\frac{ln(81)}{ln(\frac{EC_{90}\left(c,\hat{\alpha }\right)}{EC_{10}\left(c,\hat{\alpha }\right)})}=\frac{ln(81)}{ln(\frac{EC_{90}\left(\hat{c},\hat{\alpha }\right)}{EC_{10}\left(\hat{c},\hat{\alpha }\right)})}+o\left(\Delta c\right)=H\left(\hat{c},\hat{\alpha }\right)+o\left(\Delta c\right). \end{array}$$

One can assume that $\hat{\alpha }$ doesn’t change since *H* is unaffected by increasing or decreasing its value, as explained in the sections above. Also, the above analysis is carried out for *u* in a neighborhood of $EC_{10}\left(\hat{c},\hat{\alpha }\right)$ and $EC_{90}\left(\hat{c},\hat{\alpha }\right)$, hence one can assume that *u* does not vary significantly.

**Proposition 2** $H\left(\hat{c},\alpha \right)=H\left(\hat{c},\hat{\alpha }\right)+o\left(\Delta \alpha \right)$.

*Proof*: Similar to Proposition 1,

$$\begin{array}{c} {\left(\prod_{i=1}^{n}u\bar{c}\alpha_{i}+1\right)}^{1/n}=\sum_{i=1}^{n}\frac{u\bar{c}\alpha_{i}+1}{n}+o\left(\Delta \alpha \right)=u\hat{c}\hat{\alpha }+o\left(\Delta \alpha \right). \end{array}$$

Taking the *n*-th power, $\prod_{i=1}^{n}\left(u\bar{c}\alpha_{i}+1\right)={\left(u\bar{c}\bar{\alpha }+1\right)}^{n}+o\left(\Delta \alpha \right).$ In particular, for $\bar{c}=1$, $\prod_{i=1}^{n}\left(u\alpha_{i}+1\right)={\left(u\bar{\alpha }+1\right)}^{n}+o\left(\Delta \alpha \right)$. Therefore, $\eta \left(u,\hat{c},\alpha \right)=\eta \left(u,\hat{c},\hat{\alpha }\right)+o\left(\Delta \alpha \right).$ In the same way as in Proposition 1, $H(\hat{c},\alpha ).$

**Proposition 3** $H\left(c,\alpha \right)=H\left(\hat{c},\hat{\alpha }\right)+o\left(\Delta c,\Delta \alpha \right)$.

*Proof*: The first-order Taylor approximation of *H*(*c*, *α*) around $\left(\hat{c},\hat{\alpha }\right)$ can be written as

$$\begin{array}{cc} H(c,\alpha ) & =H\left(\hat{c},\hat{\alpha }\right)+\sum_{i=1}^{n}\left(c_{i}-\bar{c}\right)\frac{\partial }{\partial c_{i}}H\left(\hat{c},\hat{\alpha }\right)+\sum_{i=1}^{n}\left(\alpha_{i}-\bar{\alpha }\right)\frac{\partial }{\partial \alpha_{i}}H\left(\hat{c},\hat{\alpha }\right)+o\left(\Delta c,\Delta \alpha \right)\\ & =H\left(\hat{c},\hat{\alpha }\right)+\Delta c\cdot \nabla_{c}H\left(\hat{c},\hat{\alpha }\right)+\Delta \alpha \cdot \nabla_{\alpha }H\left(\hat{c},\hat{\alpha }\right)+o\left(\Delta c,\Delta \alpha \right). \end{array}$$

From Proposition 1, $D_{\Delta c}H\left(\hat{c},\hat{\alpha }\right)=\Delta c\cdot \nabla_{c}H\left(\hat{c},\hat{\alpha }\right)=0.$ Similarly, from Proposition 2, $D_{\Delta \alpha }H\left(\hat{c},\hat{\alpha }\right)=\Delta \alpha \cdot \nabla_{\alpha }H\left(\hat{c},\hat{\alpha }\right)=0.$ Therefore, $H\left(c,\alpha \right)=H\left(\hat{c},\hat{\alpha }\right)+o\left(\Delta c,\Delta \alpha \right)$.

### Independent system mathematical analysis

In this section, the following theorem and proposition provide mathematical analysis for the independent system showing that for *n* = 2, *H* increases when $d<\sqrt{v_{1}v_{2}}$.

**Theorem 1** *Suppose that* *f*(*u*, *z*) > 0 *is a saturating* *C*^2^ *function defined for all* *u*, *z* > 0, *such that* *f*_*u*_(*u*, *z*) > 0. *If the function*

$$\begin{array}{c} \sigma \left(u,z\right)=\frac{f_{v}\left(u,z\right)-f\left(u,z\right)f_{z}^{\infty }\left(z\right)/f^{\infty }\left(z\right)}{uf_{u}\left(u,z\right)} \end{array}$$ (8)

*is strictly increasing on u*, *then H(z) is increasing for every parameter z* > 0.

*Proof*: Let *p*(*z*) and *q*(*z*) represent the *EC*_10_ and *EC*_90_ values of the dose response respectively, that is

$$f\left(p\left(z\right),z\right)=0.10f^{\infty }\left(z\right)\;\text{and}\;f\left(q\left(z\right),z\right)=0.90f^{\infty }\left(z\right),$$

where *f*^∞^(*z*) is the maximum value of *f*(*u*, *z*). Differentiating both sides, it follows that

$$\begin{array}{c} f_{u}\left(p\left(z\right),z\right)p^{\prime }\left(z\right)+f_{v}\left(p\left(z\right),z\right)=0.10f_{z}^{\infty }\left(z\right)\;\text{and}\;f_{u}\left(q\left(z\right),z\right)q^{\prime }\left(z\right)+f_{v}\left(q\left(z\right),z\right)=0.90f_{z}^{\infty }\left(z\right). \end{array}$$

That is

$$\begin{array}{cc} p^{\prime }\left(z\right) & =\frac{-f_{z}\left(p\left(z\right),z\right)+0.10f_{z}^{\infty }\left(z\right)}{f_{u}\left(p\left(z\right),z\right)} \end{array}$$

and

$$\begin{array}{cc} q^{\prime }\left(z\right) & =\frac{-f_{z}\left(q\left(z\right),z\right)+0.90f_{z}^{\infty }\left(z\right)}{f_{u}\left(q\left(z\right),z\right)}. \end{array}$$

Recall $H\left(z\right)=\frac{\ln(81)}{\ln(q(z)/p(z))}$. Then, $\frac{dH}{dz}=\frac{-ln\left(81\right)(q^{\prime }\left(z\right)p\left(z\right)-p^{\prime }\left(z\right)q\left(z\right))}{q\left(z\right)p\left(z\right){\left(ln(\frac{q(z)}{p(z)})\right)}^{2}}$, and $\frac{dH}{dz}>0$ if and only if

$$\begin{array}{cccc} & & \frac{-ln\left(81\right)(q^{\prime }\left(z\right)p\left(z\right)-p^{\prime }\left(z\right)q\left(z\right))}{q\left(z\right)p\left(z\right){\left(ln(\frac{q(z)}{p(z)})\right)}^{2}} & >0\\ \Leftrightarrow & & p^{\prime }\left(z\right)q\left(z\right) & >q^{\prime }\left(z\right)p\left(z\right)\\ \Leftrightarrow & & \frac{-f_{z}\left(p\left(z\right),z\right)+0.10f_{z}^{\infty }\left(z\right)}{f_{u}\left(p\left(z\right),z\right)}q\left(z\right) & >\frac{-f_{z}\left(q\left(z\right),z\right)+0.90f_{z}^{\infty }\left(z\right)}{f_{u}\left(q\left(z\right),z\right)}p\left(z\right)\\ \Leftrightarrow & & \frac{f_{z}\left(p\left(z\right),z\right)-0.10f_{z}^{\infty }\left(z\right)}{p\left(z\right)f_{u}\left(p\left(z\right),z\right)} & <\frac{f_{z}\left(q\left(z\right),z\right)-0.90f_{z}^{\infty }\left(z\right)}{q\left(z\right)f_{u}\left(q\left(z\right),z\right)}\\ \Leftrightarrow & & \frac{f_{z}\left(p\left(z\right),z\right)-f\left(p\left(z\right),z\right)f_{z}^{\infty }\left(z\right)/f^{\infty }\left(z\right)}{p\left(z\right)f_{u}\left(p\left(z\right),z\right)} & <\frac{f_{z}\left(q\left(z\right),z\right)-f\left(q\left(z\right),z\right)f_{z}^{\infty }\left(z\right)/f^{\infty }\left(z\right)}{q\left(z\right)f_{u}\left(q\left(z\right),z\right)}. \end{array}$$

The last inequality follows since 0.10 = *f*(*p*(*z*), *z*)/*f*^∞^(*z*), 0.90 = *f*(*q*(*z*), *z*)/*f*^∞^(*z*). Overall, we have that

$$\begin{array}{c} \frac{dH}{dz}>0\;\text{if}\;\text{and}\;\text{only}\;\text{if}\;\sigma \left(p\left(z\right),z\right)<\sigma \left(q\left(z\right),z\right). \end{array}$$

Thus, if *σ*(*u*, *z*) is an increasing function of *u*, then $\frac{dH}{dz}>0$.

Given the dose response in (Eq 7), does *σ*(*u*, *v*) increase on *u*? Here, we provide a derivation for the associated *σ* function for the Independent dose response when *n* = 2. More specifically, we now apply the theorem to the independent model with *z* = *v*_1_.

**Proposition 4** *For the independent system in* (7), *for n* = 2, *assuming that k_i_* = *k are equal to each other*, *H*(*v*) *is increasing on v_1_ and v_2_ if* $d<\sqrt{v_{1}v_{2}}$.

*Proof*: We use the above result and show that when $d<\sqrt{v_{1}v_{2}}$, then *σ*(*u*, *v*) is increasing on *u*.

Consider *f*(*u*, *v*) as given by (7) and *σ*(*u*, *v*) from (8). For the case *n* = 2, without loss of generality let *S*_*T*_ = 1; a different value of *S*_*T*_ only re-scales the dose response and does not change *H*. Let *z* = *v*_1_. Notice that setting *z* = *v*_2_ would give the same result by symmetry. Also, let $\phi =\frac{Q_{11,v_{1}}}{Q_{11}}>0$, $Q_{I,v_{1}}=\frac{\partial Q_{I}}{\partial v_{1}}$, and $f_{u}=\frac{\partial f}{\partial u}$. It now follows that

$$\begin{array}{cccc} f(u,v) & =Q_{00}S_{00}+Q_{01}S_{01}+Q_{10}S_{10}+Q_{11}S_{11}\\ f_{v_{1}}\left(u,v\right) & =Q_{00,v_{1}}S_{00}+Q_{01,v_{1}}S_{01}+Q_{10,v_{1}}S_{10}+Q_{11,v_{1}}S_{11} & f_{v_{1}}^{\infty }\left(v\right) & =Q_{11,v_{1}}\\ f_{u}\left(u,v\right) & =Q_{00}S_{00}^{\prime }+Q_{01}S_{01}^{\prime }+Q_{10}S_{10}^{\prime }+Q_{11}S_{11}^{\prime } & f^{\infty }\left(v\right) & =Q_{11}. \end{array}$$

Then,

$$\begin{array}{cc} \sigma (u,v_{1}) & =\frac{f_{v_{1}}\left(u,v_{1}\right)-f\left(u,v_{1}\right)f_{v_{1}}^{\infty }\left(v_{1}\right)/f^{\infty }\left(v_{1}\right)}{uf_{u}\left(u,v_{1}\right)}\\ & =\frac{Q_{00,v_{1}}S_{00}+Q_{01,v_{1}}S_{01}+Q_{10,v_{1}}S_{10}+Q_{11,v_{1}}S_{11}-\left(Q_{00}S_{00}+Q_{01}S_{01}+Q_{10}S_{10}+Q_{11}S_{11}\right)\frac{Q_{11,v_{1}}}{Q_{11}}}{u(Q_{00}S_{00}^{\prime }+Q_{01}S_{01}^{\prime }+Q_{10}S_{10}^{\prime }+Q_{11}S_{11}^{\prime })}\\ & =\frac{\frac{Q_{10,v_{1}}ku}{{\left(u+k\right)}^{2}}+\frac{Q_{01,v_{1}}ku}{{\left(u+k\right)}^{2}}+\frac{Q_{11,v_{1}}u^{2}}{{\left(u+k\right)}^{2}}-(Q_{00}\frac{k^{2}}{{\left(u+k\right)}^{2}}+Q_{10}\frac{ku}{{\left(u+k\right)}^{2}}+Q_{01}\frac{ku}{{\left(u+k\right)}^{2}}+Q_{11}\frac{u^{2}}{{\left(u+k\right)}^{2}})\phi }{u(Q_{00}\frac{-2k^{2}}{{\left(u+k\right)}^{3}}+Q_{10}\frac{k(k-u)}{{\left(u+k\right)}^{3}}+Q_{01}\frac{k(k-u)}{{\left(u+k\right)}^{3}}+Q_{11}\frac{2uk}{{\left(u+k\right)}^{3}})}\\ & =\frac{\frac{1}{{\left(u+k\right)}^{2}}}{\frac{u}{{\left(u+k\right)}^{3}}}\frac{kuQ_{10,v_{1}}+kuQ_{01,v_{1}}+u^{2}Q_{11,v_{1}}-\phi (k^{2}Q_{00}+ukQ_{01}+ukQ_{10}+u^{2}Q_{11})}{-2k^{2}Q_{00}+k\left(k-u\right)Q_{10}+k\left(k-u\right)Q_{01}+2ukQ_{11}}\\ & =\frac{u+k}{u}\frac{ku\left(Q_{10,v_{1}}+Q_{01,v_{1}}\right)-\phi k^{2}Q_{00}-uk\phi \left(Q_{10}+Q_{01}\right)}{k^{2}\left(-2Q_{00}+Q_{10}+Q_{01}\right)+uk\left(-Q_{10}-Q_{01}+2Q_{11}\right)}\\ & =\frac{u+k}{u}\frac{u\left(Q_{10,v_{1}}+Q_{01,v_{1}}-\phi Q_{10}-\phi Q_{01}\right)-\phi kQ_{00}}{k\left(Q_{10}+Q_{01}-2Q_{00}\right)+u\left(2Q_{11}-Q_{10}-Q_{01}\right)}\\ & =-\frac{u+k}{u}\frac{u\left(\phi Q_{10}+\phi Q_{01}-Q_{10,v_{1}}-Q_{01,v_{1}}\right)+\phi kQ_{00}}{u\left(2Q_{11}-Q_{10}-Q_{01}\right)+k\left(Q_{10}+Q_{01}-2Q_{00}\right)}\\ & =-\frac{u+k}{C_{3}u+C_{4}}\frac{C_{1}u+C_{2}}{u}, \end{array}$$

where

$$\begin{array}{cccc} C_{1} & =\phi Q_{10}+\phi Q_{01}-Q_{10,v_{1}}-Q_{01,v_{1}} & C_{3} & =2Q_{11}-Q_{01}-Q_{10}>0\\ C_{2} & =\phi kQ_{00}>0 & C_{4} & =k(Q_{10}+Q_{01}-2Q_{00})>0. \end{array}$$

Let $\tau_{1}=\frac{u+k}{C_{3}u+C_{4}}$ and $\tau_{2}=-\frac{C_{1}u+C_{2}}{u}$, so that *σ* = *τ*_1_*τ*_2_. Note that *τ*_2_ is a strictly increasing function on *u* since $\tau_{2}=-C_{1}-\frac{C_{2}}{u}$.

In the following text, we show that *τ*_1_ is also strictly increasing on *u* if and only if $d<\sqrt{v_{1}v_{2}}$. To see this, notice that *τ*_1_ is strictly increasing if and only if $\frac{k}{C_{4}}<\frac{1}{C_{3}}$, which is equivalent to *C*_3_ *k* < *C*_4_. This is equivalent to

$$\begin{array}{cc} k(2Q_{11}-Q_{01}-Q_{10}) & <k(Q_{01}+Q_{10}-2Q_{00})\\ 2Q_{11}-Q_{01}-Q_{10} & <Q_{01}+Q_{10}-2Q_{00}\\ 0 & <Q_{01}+Q_{10}-Q_{00}-Q_{11}\\ 0 & <\frac{v_{2}}{d+v_{2}}+\frac{v_{1}}{d+v_{1}}-\frac{1}{d+1}-\frac{v_{1}v_{2}}{d+v_{1}v_{2}}\\ 0 & <v_{2}\left(d+v_{1}\right)\left(d+1\right)\left(d+v_{1}v_{2}\right)+v_{1}\left(d+v_{2}\right)\left(d+1\right)\left(d+v_{1}v_{2}\right)\\ & -\left(d+v_{2}\right)\left(d+v_{1}\right)\left(d+v_{1}v_{2}\right)-v_{1}v_{2}\left(d+1\right)\left(d+v_{2}\right)\left(d+v_{1}\right)\\ 0 & <v_{2}\left(d+1\right)\left(d+v_{1}\right)[d+v_{1}v_{2}-v_{1}\left(d+v_{2}\right)]\\ & +\left(d+v_{2}\right)\left(d+v_{1}v_{2}\right)[v_{1}\left(d+1\right)-\left(d+v_{1}\right)]\\ 0 & <v_{2}d\left(d+1\right)\left(d+v_{1}\right)\left(1-v_{1}\right)+d\left(d+v_{2}\right)\left(d+v_{1}v_{2}\right)\left(v_{1}-1\right)\\ 0 & <\left(v_{1}-1\right)[-v_{2}\left(d+1\right)\left(d+v_{1}\right)+\left(d+v_{2}\right)\left(d+v_{1}v_{2}\right)]\\ 0 & <\left(d+v_{2}\right)\left(d+v_{1}v_{2}\right)-v_{2}\left(d+1\right)\left(d+v_{1}\right)\\ 0 & <d^{2}+dv_{1}v_{2}+dv_{2}+v_{1}v_{2}^{2}-v_{2}d^{2}-v_{1}v_{2}d-v_{2}d-v_{1}v_{2}\\ 0 & <d^{2}+v_{1}v_{2}^{2}-v_{2}d^{2}-v_{1}v_{2}\\ 0 & <\left(v_{2}-1\right)\left(v_{1}v_{2}-d^{2}\right)\\ 0 & <(v_{1}v_{2}-d^{2})\\ d^{2} & <v_{1}v_{2}\\ d & <\sqrt{v_{1}v_{2}}. \end{array}$$

As long as $d<\sqrt{v_{1}v_{2}}$, it follows that *τ*_1_ is an increasing function on *u* making *σ* the product of two increasing functions and thus, *σ* is increasing on *u*.

**Theorem 2** *Suppose n* = 2. *If Q*_01_ > 1/2 *and Q*_10_ > 1/2, *then H is increasing as a function of* *v*_1_ *and* *v*_2_.

*Proof*: If *Q*_01_ > 1/2 and *Q*_10_ > 1/2, it follows that $\frac{v_{1}}{d+v_{1}}>\frac{1}{2}$ and $\frac{v_{2}}{d+v_{2}}>\frac{1}{2}.$

Then,

$$\begin{array}{cccccc} v_{1} & >\frac{1}{2}\left(d+v_{1}\right) & \;\text{and}\; & v_{2} & & >\frac{1}{2}\left(d+v_{2}\right)\\ 2v_{1} & >d+v_{1} & \;\text{and}\; & 2v_{2} & & >d+v_{2}\\ v_{1} & >d & \;\text{and}\; & v_{2} & & >d. \end{array}$$

Thus, *d*^2^ < *v*_1_ *v*_2_ and so $d<\sqrt{v_{1}v_{2}}$. Thus, by Proposition (4), *H* is increasing as a function of *v*_1_ and *v*_2_.

The point where $d=\sqrt{v_{1}v_{2}}$ (the dotted line in Fig 4c) corresponds to the case where the conformational free energy contribution contributed by the singly modified forms is equal to exactly half of that contributed by the doubly modified forms. It can be shown that this situation (which we shall call the ‘linear regime’) results in a dose response curve with a Hill number of 1. If *v*_1_ and/or *v*_2_ are then increased so that $\sqrt{v_{1}v_{2}}$ (the region to the right of the dotted line in Fig 4c) becomes greater than *d*, the singly modified forms now have more than half the conformational free energy contribution of the doubly modified forms, and the Hill number increases. The Hill number will continue to increase until the two singly modified forms, collectively, contribute exactly the same conformational free energy contribution as the doubly modified form. At this point, the system is in the ‘+ regime’, where modification of one, the other, or both sites lead to the same level of activation.

On the other hand, if $d>\sqrt{v_{1}v_{2}}$, then the system is closer to the ‘both or none regime’, where efficient activation only occurs when both sites are modified. Here, increasing *v*_1_ or *v*_2_ reduces the Hill number by pushing the system away from ‘both or none’ and closer to ‘linear’.

## Discussion

In a protein with multiple ligand binding sites, the individual sites can differ from each other in two ways: in their microscopic ligand binding affinity, and in the energetic contribution they make, once bound or modified, to functional outcomes such as a ligand-induced conformational change in the bound protein. Likewise, for a protein that is post-translationally modified on multiple sites, the individual sites may have different modification efficiencies, and may also, independently, make differential contributions to downstream functional consequences once modified. For example, in the case of phosphorylation, the amino acid sequence around the target phosphoacceptor residue can substantially influence the efficiency of phosphorylation by the relevant kinase, as well as the efficiency of dephosphorylation by cellular phosphatases [34]. Such tuning of the steady-state level of site modification is biochemically distinct and clearly separable from the effects that the phosphorylation of that site will have on the conformation of the target molecule, its ability to bind other macromolecules, etc. [35–37].

Previously, Enciso and Ryerson [8] asked the question “how can the microscopic ligand binding affinities (a.k.a. modification efficiencies) be tuned if the goal is to maximize ultrasensitivity?” Interestingly, they found that ultrasensitivity was maximal when the microscopic affinities were balanced. For instance, for a protein with 4 ligand binding sites, ultrasensitivity was maximized when all 4 sites had exactly the same ligand binding affinity. For a protein with 4 phosphorylation sites, ultrasensitivity was maximized when all 4 sites had the same phosphorylation/dephosphorylation efficiency.

Here we examined how differential energetic contributions of the sites might affect the performance objective of ultrasensitivity. We considered a simple model in which binding/modification promotes a conformational change that flips the modified molecule from an inactive to an active state; this example is readily extended to other known consequences of ligand binding or post-translational modification. We generalized the classic allosteric MWC model to allow for differences in the energetic contributions for any number of distinct sites. We also considered an independent modification model that does not assume allostery or cooperativity among sites. For the generalized MWC system, we found that ultrasensitivity generally increased when the energetic contribution (i.e., the conformational free energy contribution) of one or more of the sites was strengthened. Here, ‘strengthened’ means that the conformational free energy contribution became more negative; this results in the corresponding activation parameter c becoming smaller. Furthermore, we found that there was no benefit derived from balancing the conformational free energies, nor any penalty for unbalancing them. Our results have implications for understanding the potential trajectories that can be pursued by a protein under selective pressure to increase the ultrasensitivity of its response to modification.

Regarding our finding that decreasing the activation parameter *c*_*i*_ of individual sites has a strong tendency to increase the Hill coefficient, this result is analogous to work by Rubin and Changeux [38]. In Fig 2 of that work the authors illustrate computationally that for fixed parameter values of the MWC model, decreasing *c* leads to an increase in a different version of the Hill coefficient. In our paper we are able to consider individual sites, rather than all sites together, so our result is in a sense a generalization of that shown in [38].

Despite the fact that there is no penalty associated with the conformational free energies being unbalanced, our model nevertheless suggests a factor that may tend to lead to roughly balanced conformational free energies: diminishing returns. Successive, equal-valued improvements of conformational free energy contribution are diminishing with respect to their effect on ultrasensitivity. That is, changes that are of equal magnitude to previous changes increase ultrasensitivity by a smaller amount than the previous changes did. Furthermore, changes to weaker sites increase ultrasensitivity more dramatically than equivalent changes to stronger sites. Eventually, the marginal increase in ultrasensitivity caused by additional improvements to conformational free energy contribution becomes negligible. At this point it can be argued that a zone of effective neutrality has been reached, where the probability of fixation of a new mutation that incrementally improves ultrasensitivity will be essentially indistinguishable from the probability of fixation of a neutral mutation [39]. At this point, substantial improvement to ultrasensitivity can only arise if the molecule evolves an additional site.

There is an additional factor that may further promote the balancing of conformational free energies. Using both computational and mathematical analysis, we showed that when the sites are at least moderately active, the ultrasensitivity is mostly dependent on the mean of the activation parameters and is largely independent from their variance (Fig 3a). Since increasing the variance of the activation parameters tends to be associated with weaker (less negative) total conformational free energy contribution, a prediction is that the sites tend to have roughly equal activation parameters. This can be implemented e.g. by bulk electrostatic mechanisms, which are commonly found experimentally [23, 24, 40].

These considerations lead to a prediction that conformational free energies will be roughly balanced, with a kcal/mol value roughly equal to the point where ultrasensitivity starts to level out substantially. As shown in Fig 3b and 3c and Table 1 and S1 Table, this “leveling out point” is roughly between -2 to -4 kcal/mol per site, depending on the number of sites *n* and the level of basal activation (which is determined by the parameter *L*). This range of -2 to -4 kcal/mol does assume that the efficiency of modification is roughly constant across all sites, but is otherwise surprisingly independent of other parameters. For example, the range found changed very little upon variation of the value of *L* from 30 to 10,000, therefore covering most biochemically realistic values for this constant. The range of approximately -2 to -4 kcal/mol corresponds to activation coefficient (*c*) values between approximately 0.05 and 0.001. Such *c* values are all within the range reported for classic “MWC enzymes” such as threonine deaminise, glucose-6-phosphate deaminase, aspartate transcarbamoylase and glyceraldehyde-3-phosphate dehydrogenase [41–44]. Moreover, with regard to phosphorylation, the effect of a single phosphate on conformation [25], protein-protein binding [26] or protein-membrane binding [23, 24] has been estimated to be about 2 kcal/mol.

We also showed (Fig 3d) that when the number of sites is large, and a hypothetical maintenance cost per site is included (such as might arise from rapid phosphorylation-dephosphorylation cycles [31, 32]), an optimal strategy to maximize ultrasensitivity can be to focus on a subset of the sites, and essentially keep the other sites silent. In such cases, evolving another site is not a viable strategy to increase ultrasensitivity, and it can be argued that there is an optimal number of functional sites that will maximize benefit (ultrasensitivity) while containing cost.

This analysis applies for other forms of multisite modification other than phosphorylation such as ligand binding, methylation, acetylation, etc. When the multisite target molecule has a symmetric structure (such as hemoglobin which is a tetramer), one can assume that the conformational free energy contribution is similar across all sites. In this sense the current study is most relevant when the target structure is more heterogeneous, such as in the case of phosphorylation. Although phosphorylation consumes energy and is not thermodynamically closed, the MWC model is still a popular model to describe it [8, 45, 46]. It is also mathematically more amenable than the non-allosteric, independent model that we also included for completeness.

Work by Kafri et al [19] has previously studied a mathematical model of chaperon-containing TCP-1 protein that has several sites with different ATP binding affinities. This system can provide very interesting parallels with our framework. Their mathematical model shows that when a protein has multiple sites with different ligand affinities, the Hill coefficient can be reduced leading to apparent negative cooperativity. We do observe a similar effect (see eg Fig 3), although that model has important differences such as variability in modification affinity rather than conformational free energy contribution.

The analysis in this manuscript is limited to systems in equilibrium, i.e. the long term response to a constant input. For non-equilibrium systems, and in situations where energy is used, recent work by Estrada et al. [47] shows that one can obtain a larger Hill coefficient. The authors use techniques similar to kinetic proofreading, which can give rise to large response differences given small differences in ligand affinity. A full discussion of non-equilibrium dynamics is however outside of the scope of our work.

Many dose response curves for allosterically-regulated proteins can be well-modeled by the standard MWC model. Our goal in generalizing the MWC model was to explore the qualitative theoretical consequences of allowing the conformational free energies of different sites to vary, and not to make a tool for empirical fitting to data. On this point, however, it should be noted that Stefan et al. [48] have shown how an extended MWC model such as the one developed here can be used in parameter estimation, and experimental methods to measure MWC parameters are constantly improving [49–51]. Another useful recent tool is a method developed by Gruber et al. [52] which facilitates the determination of parameters in the MWC model, by finding a theoretical relationship between the Hill coefficient and model parameters.

## Supporting information

S1 Fig Statistical weights of MWC modification states for n = 3. Table of each possible modification state in the generalized MWC system above when *n* = 3 and the corresponding statistical weight of that state. When *n* = 3, the associated partition function *Z* = 1 + *α*_1_ + *α*_2_ + *α*_3_ + *α*_1_*α*_2_ + *α*_1_*α*_3_ + *α*_3_*α*_3_ + *α*_1_*α*_2_*α*_3_ + *L* + *α*_1_*c*_1_*L* + *α*_2_*c*_2_*L* + *α*_3_*c*_3_*L* + *α*_1_*α*_2_*c*_1_*c*_2_*L* + *α*_1_*α*_3_*c*_1_*c*_3_*L* + *α*_2_*α*_3_*c*_2_*c*_3_*L* + *α*_1_*α*_2_*α*_3_*c*_1_*c*_2_*c*_3_*L*.
(TIF)

S2 FigUltrasensitivity of MWC system.Heat maps for H when *c*1, *c*2 ∈ [10^−4^, 0.9] with *L* = 1000 and $\alpha_{i}=\bar{\alpha }=1$ and (a) *n* = 2, (b) *n* = 4, *c*_*i*_ = 0.01 for *i* ≥ 3, similarly with (c) *n* = 8. These figures are the same data points from Fig 2 in a linear scale.(TIF)

S3 FigParameters and H in MWC.(a) Scatter plot for *H* and the arithmetic mean of *c* where *c*_*i*_ are independently and logarithmically chosen from [10^−4^, 0.9], *L* = 1000, and *α*_*i*_ are independently and logarithmically chosen from [0.1, 10] for *n* = 2, 3, 4, 8. (b) *H* from (a) with the coefficient of variation (CV) along the x-axis. (c) Scatter plot for *H* when increasing total conformational free energy with *c*_*i*_ ∈ [10^−4^, 0.9], *L* = 30 and $\alpha_{i}=\bar{\alpha }$ for *n* = 2, 3, 4, 8. (d) Scatter plot for *H* for when *L* = 10, 000. (e) Proportion of 10000 parameter sets in which *H* decreased when a *c*_*i*_ is marginally increased for different values *L* and *n*. (f) *H* values for key scenarios. When a target molecule has 3 sites, *H* = 2.40 when $\alpha_{i}=\bar{\alpha }=1$, *L* = 1000, and *c*_*i*_ = 0.01. Adding a site with *c*_4_ = 1 will yield the same *H*. However, if *c*_4_ = 0.01, *H* = 3.08.(TIF)

S4 FigUltrasensitivity and total conformational free energy in MWC.Scatter plots for ultrasensitivity when increasing total conformational free energy with *c*_*i*_ ∈ [10^−4^, 0.9], *L* = 1000 and $\alpha_{i}=\bar{\alpha }=1$ for *n* = 2, 3, 4, 8 and 10000 points. Ultrasensitivity is measured with (a) a non-linear regression fit to the Hill function $f=\frac{x^{H}}{k^{H}+x^{H}}$, where *H* is the Hill number labeled *H*_*Fit*_ and (b) a generalized Levitzki derivation for ultrasensitivity [30] as *H*_*Lev*_ = 4 * *EC*_50_ * *f*′(*EC*_50_, *α*, *c*) where *f*′(*EC*_50_, *c*, *α*) is the derivative of the dose response function evaluated at the *EC*_50_, the effective enzyme/ligand concentration at which there is a 50% maximal protein response, labeld *H*_*Lev*_. We can consider *H*_*Lev*_ as the sensitivity at 50% maximal response. *EC*_50_ was found with the standard MatLab fzero solver and the derivative with diff after normalizing to the *f*^∞^(*c*).(TIF)

S5 FigUltrasensitivity and total conformational free energy in MWC with Maintenance costs.Scatter plots for ultrasensitivity when increasing total conformational free energy with *c*_*i*_ ∈ [10^−4^, 0.9], *L* = 1000 and $\alpha_{i}=\bar{\alpha }=1$ for *n* = 2, 3, 4, 8 and a maintenance cost of (a) *M*_*c*_ = 2, (b) *M*_*c*_ = 4 (from Fig 3) and (c) *M*_*c*_ = 8. The Ste5 data point is added for illustration purposes with the same maintenance cost for each of the 8 phosphorylation sites.(TIF)

S1 TableUltrasensitivity at knee.Ultrasensitivity as measured by the Goldbeter-Koshland formula described in Eq (2) along with the approximated knee of curves similar to those in Fig 3c for fixed values of *L* and *n*. Parameters $\alpha_{i}=\bar{\alpha }=1$.(TIF)
