# Supplementary figures and images for: Effect of magnitude and variability of energy of activation in multisite ultrasensitive biochemical processes
Source: PLoS Comput Biol. 2020 Aug 6;16(8):e1007966. doi: 10.1371/journal.pcbi.1007966 (PMC7444825; doi:10.1371/journal.pcbi.1007966)

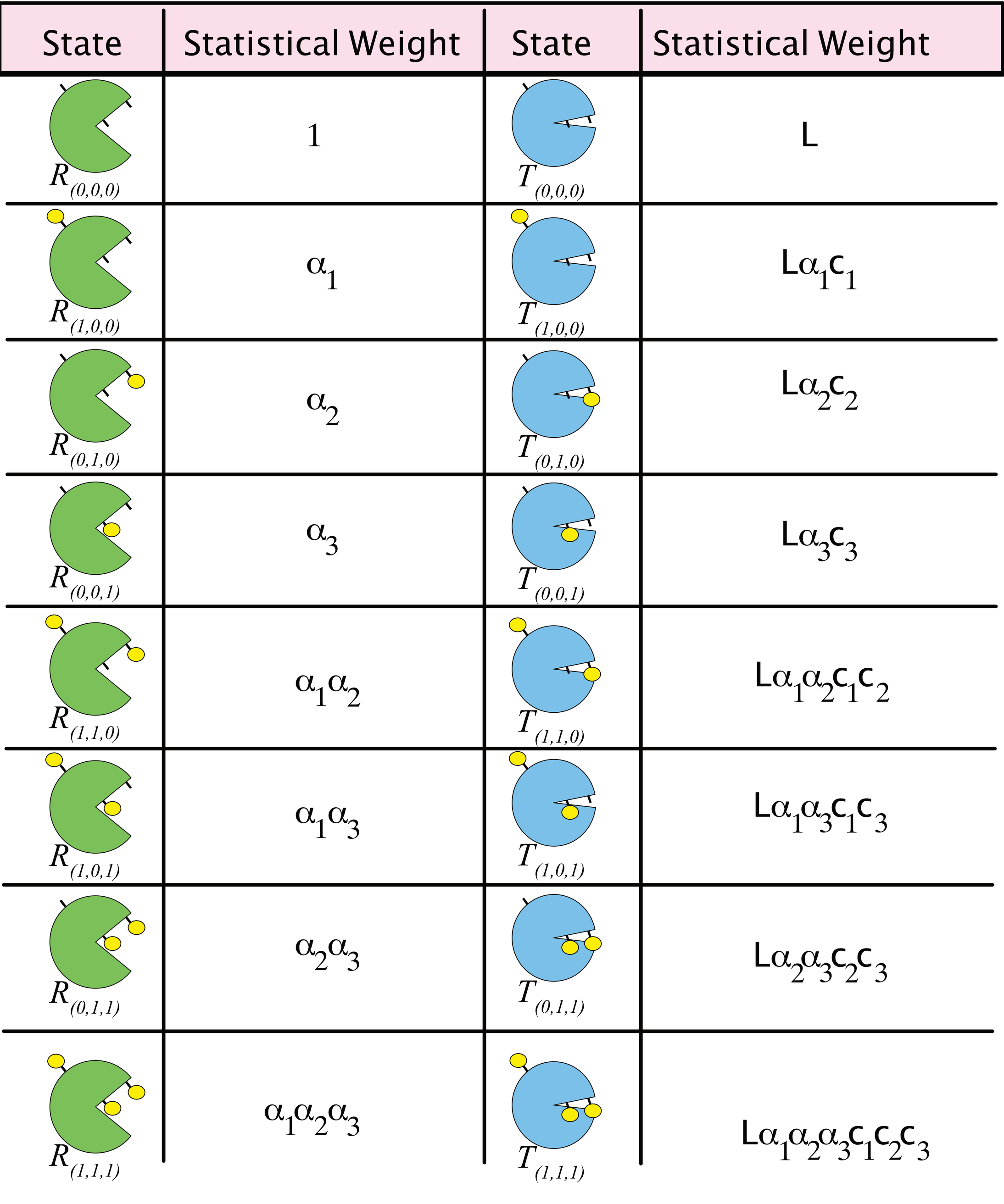

Supplement: S1 Fig — Table of each possible modification state in the generalized MWC system above when n = 3 and the corresponding statistical weight of that state. When n = 3, the associated partition function Z = 1 + α1 + α2 + α3 + α1α2 + α1α3 + α3α3 + α1α2α3 + L + α1c1L + α2c2L + α3c3L + α1α2c1c2L + α1α3c1c3L + α2α3c2c3L + α1α2α3c1c2c3L. (TIF) [file pcbi.1007966.s001.tif]

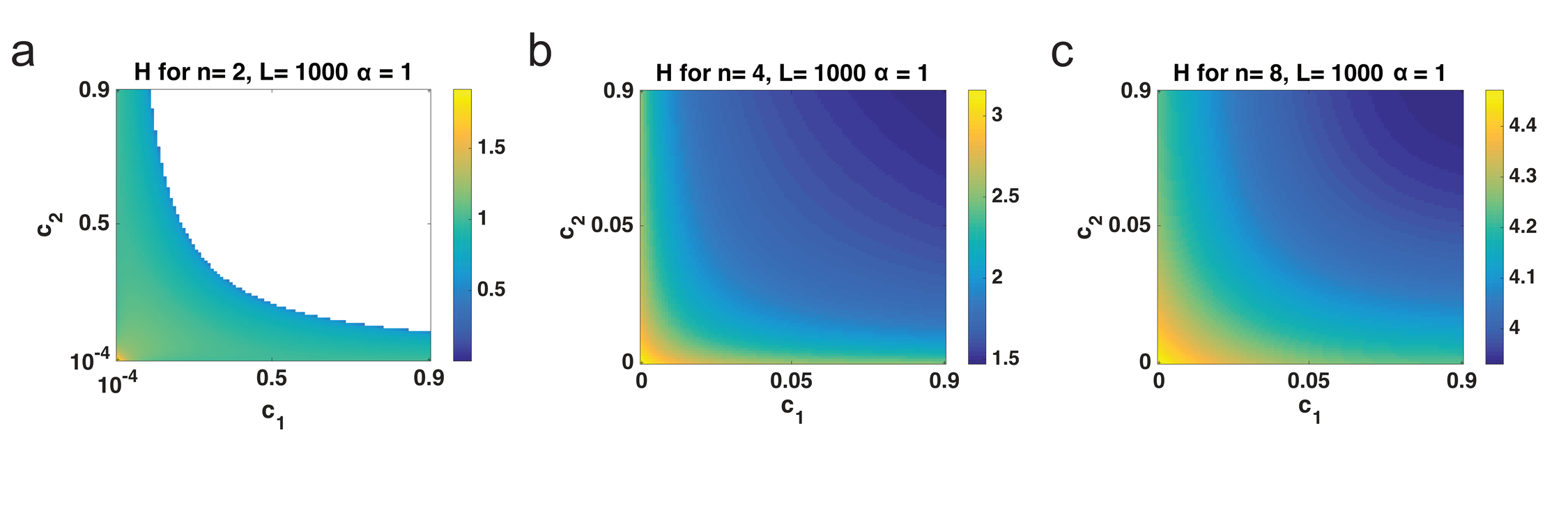

Supplement: S2 Fig — Heat maps for H when c1, c2 ∈ [10−4, 0.9] with L = 1000 and αi=α¯=1 and (a) n = 2, (b) n = 4, ci = 0.01 for i ≥ 3, similarly with (c) n = 8. These figures are the same data points from Fig 2 in a linear scale. (TIF) [file pcbi.1007966.s002.tif]

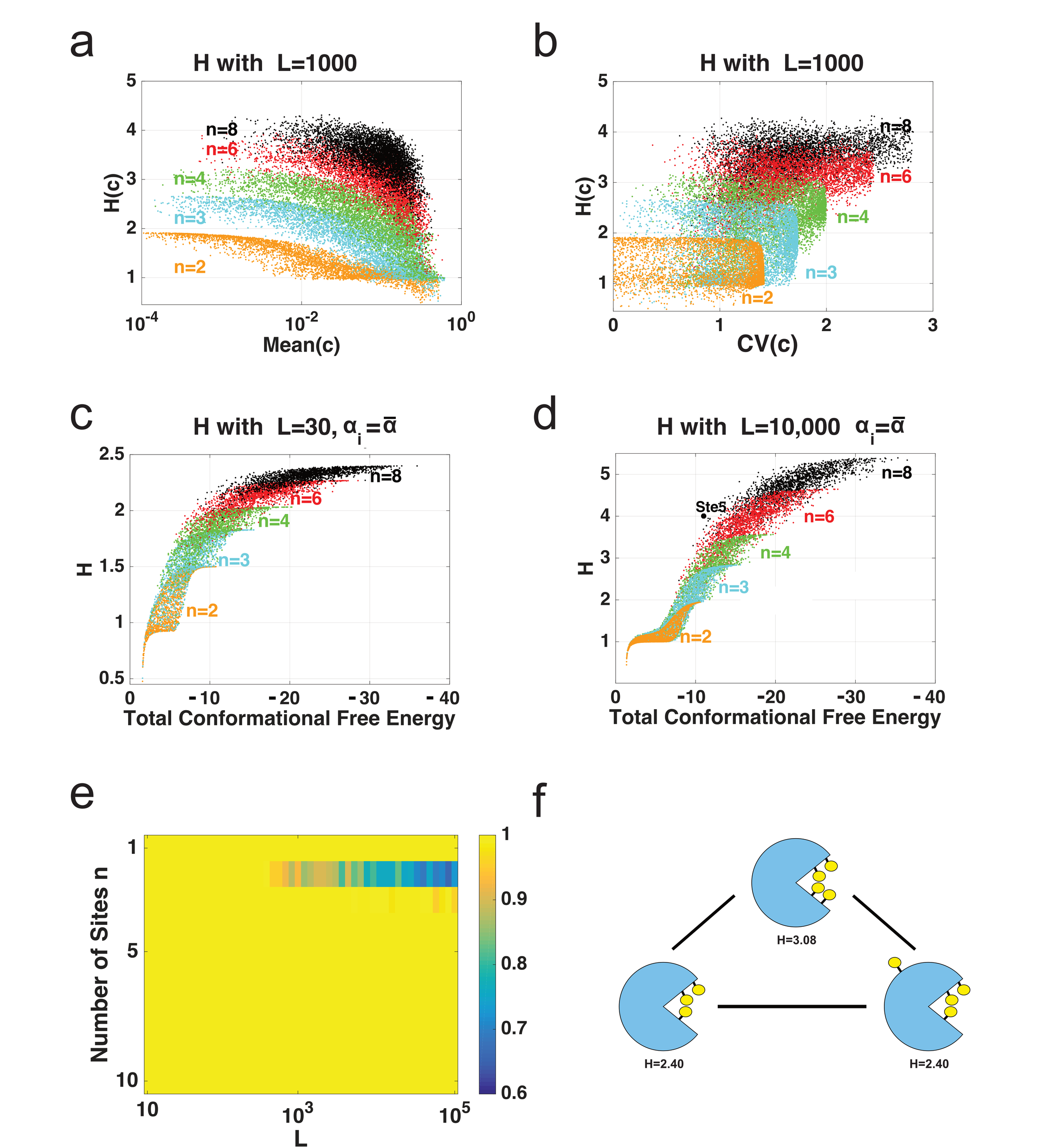

Supplement: S3 Fig — (a) Scatter plot for H and the arithmetic mean of c where ci are independently and logarithmically chosen from [10−4, 0.9], L = 1000, and αi are independently and logarithmically chosen from [0.1, 10] for n = 2, 3, 4, 8. (b) H from (a) with the coefficient of variation (CV) along the x-axis. (c) Scatter plot for H when increasing total conformational free energy with ci ∈ [10−4, 0.9], L = 30 and αi=α¯ for n = 2, 3, 4, 8. (d) Scatter plot for H for when L = 10, 000. (e) Proportion of 10000 parameter sets in which H decreased when a ci is marginally increased for different values L and n. (f) H values for key scenarios. When a target molecule has 3 sites, H = 2.40 when αi=α¯=1, L = 1000, and ci = 0.01. Adding a site with c4 = 1 will yield the same H. However, if c4 = 0.01, H = 3.08. (TIF) [file pcbi.1007966.s003.tif]

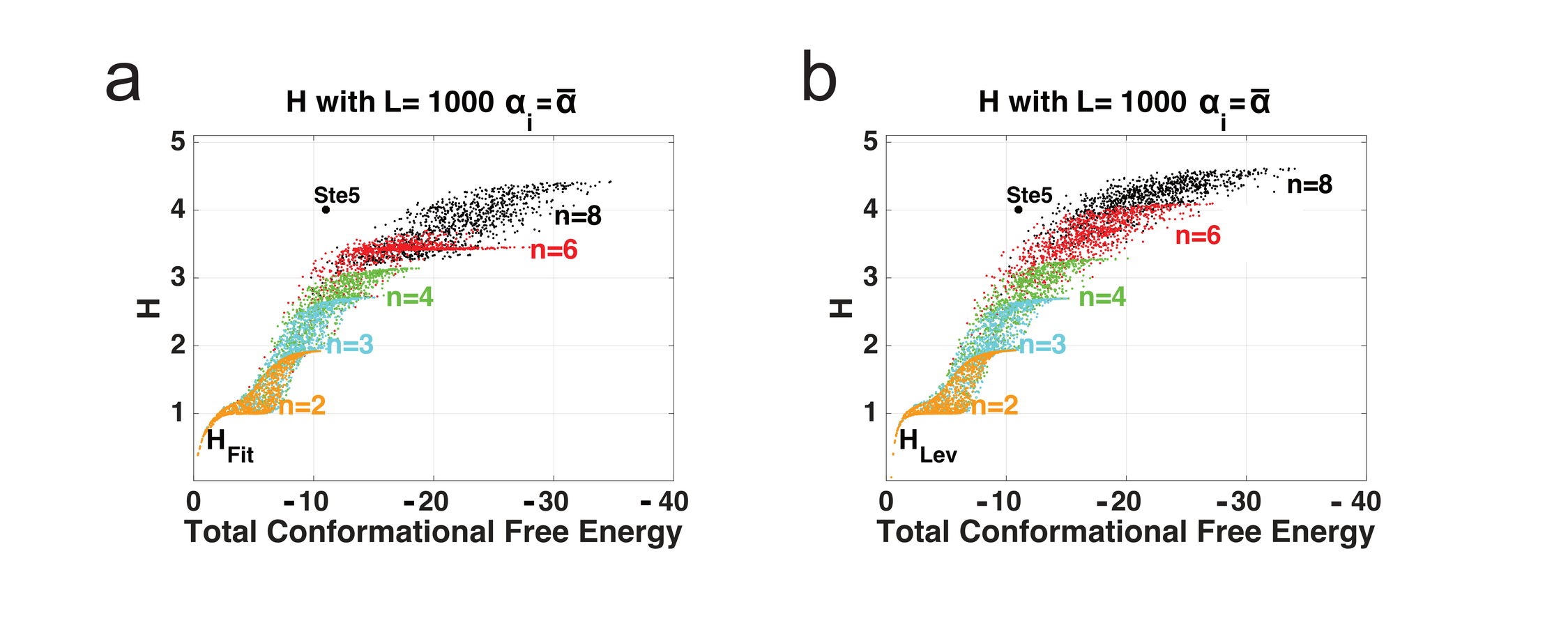

Supplement: S4 Fig — Scatter plots for ultrasensitivity when increasing total conformational free energy with ci ∈ [10−4, 0.9], L = 1000 and αi=α¯=1 for n = 2, 3, 4, 8 and 10000 points. Ultrasensitivity is measured with (a) a non-linear regression fit to the Hill function f=xHkH+xH, where H is the Hill number labeled HFit and (b) a generalized Levitzki derivation for ultrasensitivity [30] as HLev = 4 * EC50 * f′(EC50, α, c) where f′(EC50, c, α) is the derivative of the dose response function evaluated at the EC50, the effective enzyme/ligand concentration at which there is a 50% maximal protein response, labeld HLev. We can consider HLev as the sensitivity at 50% maximal response. EC50 was found with the standard MatLab fzero solver and the derivative with diff after normalizing to the f∞(c). (TIF) [file pcbi.1007966.s004.tif]

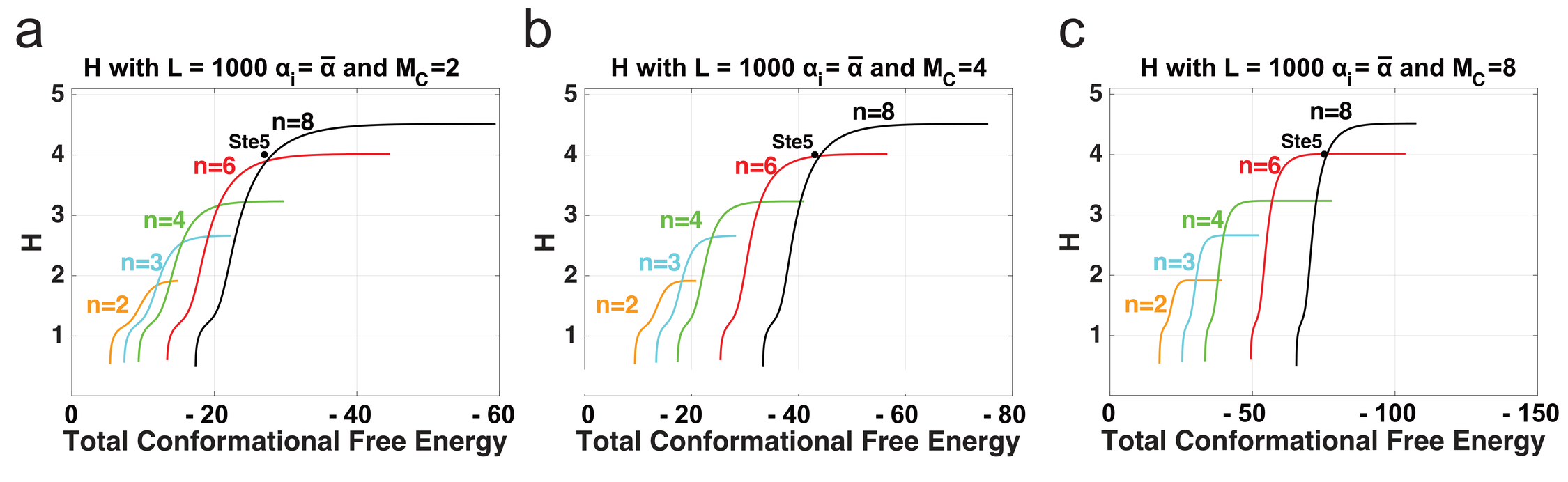

Supplement: S5 Fig — Scatter plots for ultrasensitivity when increasing total conformational free energy with ci ∈ [10−4, 0.9], L = 1000 and αi=α¯=1 for n = 2, 3, 4, 8 and a maintenance cost of (a) Mc = 2, (b) Mc = 4 (from Fig 3) and (c) Mc = 8. The Ste5 data point is added for illustration purposes with the same maintenance cost for each of the 8 phosphorylation sites. (TIF) [file pcbi.1007966.s005.tif]

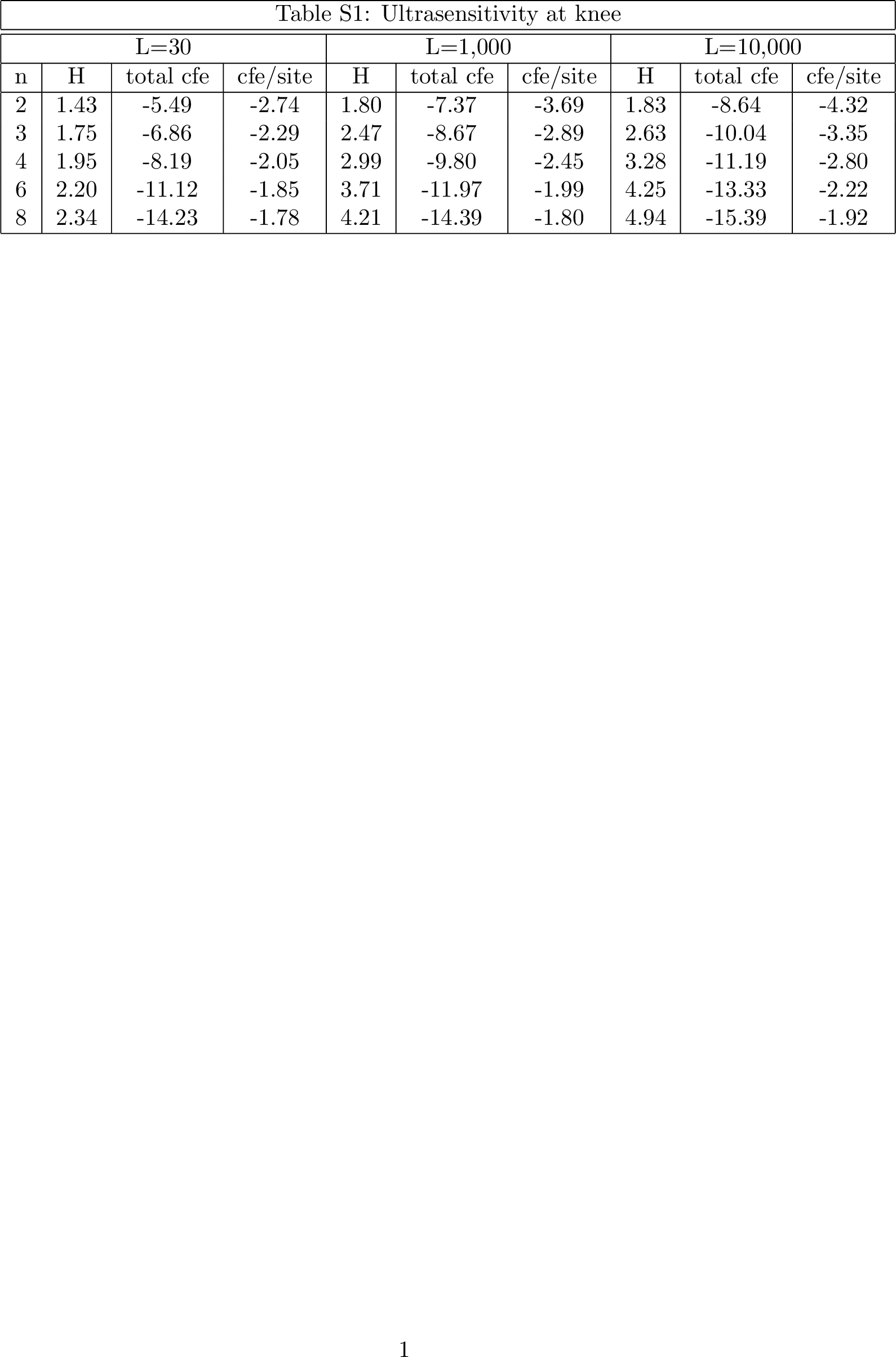

Supplement: S1 Table — Ultrasensitivity as measured by the Goldbeter-Koshland formula described in Eq (2) along with the approximated knee of curves similar to those in Fig 3c for fixed values of L and n. Parameters αi=α¯=1. (TIF) [file pcbi.1007966.s006.tif]
